# IGF-1 modulates gene expression of proteins involved in inflammation, cytoskeleton, and liver architecture

**DOI:** 10.1007/s13105-016-0545-x

**Published:** 2017-01-26

**Authors:** VJ Lara-Diaz, I Castilla-Cortazar, I Martín-Estal, M García-Magariño, GA Aguirre, JE Puche, RG de la Garza, LA Morales, U Muñoz

**Affiliations:** 1 Escuela de Medicina, Tecnologico de Monterrey, Avenida Morones Prieto No. 3000 Pte. Col. Los Doctores, 64710 Monterrey, Nuevo León Mexico; 2 Fundacion de Investigacion HM Hospitales, Madrid, Spain; 3 Department of Medical Physiology, School of Medicine, Universidad San Pablo-CEU, Madrid, Spain

**Keywords:** IGF-1, Gene expression, Cytoskeleton, Tight junctions, Hepatocytes, Extracellular matrix

## Abstract

Even though the liver synthesizes most of circulating IGF-1, it lacks its receptor under physiological conditions. However, according to previous studies, a damaged liver expresses the receptor. For this reason, herein, we examine hepatic histology and expression of genes encoding proteins of the cytoskeleton, extracellular matrix, and cell-cell molecules and inflammation-related proteins. A partial IGF-1 deficiency murine model was used to investigate IGF-1’s effects on liver by comparing wild-type controls, heterozygous igf1^+/−^, and heterozygous mice treated with IGF-1 for 10 days. Histology, microarray for mRNA gene expression, RT-qPCR, and lipid peroxidation were assessed. Microarray analyses revealed significant underexpression of *igf1* in heterozygous mice compared to control mice, restoring normal liver expression after treatment, which then normalized its circulating levels. IGF-1 receptor mRNA was overexpressed in Hz mice liver, while treated mice displayed a similar expression to that of the controls. Heterozygous mice showed overexpression of several genes encoding proteins related to inflammatory and acute-phase proteins and underexpression or overexpression of genes which coded for extracellular matrix, cytoskeleton, and cell junction components. Histology revealed an altered hepatic architecture. In addition, liver oxidative damage was found increased in the heterozygous group. The mere IGF-1 partial deficiency is associated with relevant alterations of the hepatic architecture and expression of genes involved in cytoskeleton, hepatocyte polarity, cell junctions, and extracellular matrix proteins. Moreover, it induces hepatic expression of the IGF-1 receptor and elevated acute-phase and inflammation mediators, which all resulted in liver oxidative damage.

**Electronic supplementary material:**

The online version of this article (doi:10.1007/s13105-016-0545-x) contains supplementary material, which is available to authorized users.

## Introduction

The liver is the main source of circulating insulin-like growth factor-1 (IGF-1) (more than 75%). It is produced following growth hormone (GH) endocrine stimulus. IGF-1 is a 70-amino acid hormone with effects on almost every tissue and organ [27–29]. However, the liver is not a target organ for this hormone as liver cells do not express the receptor under physiological conditions. Additionally, exceptions to this are hepatic regeneration (when injury to the organ has occurred), fetal liver, and malignant transformation of the cells (i.e., when cells become tumorous) [3, 29, 33, 34, 50].

Pituitary-secreted GH and liver-produced IGF-1 establish a negative feedback mechanism to maintain a controlled GH/IGF-1 axis [2, 7]. Circulating IGF-1 can be found in its biologically active free form; it is however mainly bound to proteins (IGF-1 binding proteins, IGFBPs), especially to IGFBP-3 [24] to prolong their half-life from minutes to hours. Since IGF-1 has a wide range of physiological roles, its activity must be strictly controlled, where IGFBPs play their part. These binding proteins also help to modulate the interaction between IGF-1 and its receptor (IGF-1R), thereby indirectly controlling IGF-1 biological activity [15]. Moreover, IGFBPs possess IGF-1-independent actions mediated by their own membrane or intracellular receptors [15].

The variety of IGF-1 activities can be partly summarized as cell proliferation and differentiation; tissue growth and development; insulin-like activity; anti-inflammatory; and antioxidant, mitochondrial protection, and prosurvival/antiaging. Moreover, its deficiency has been initially related to different pathologies, such as Laron’s syndrome, intrauterine growth restriction (IUGR), liver cirrhosis, metabolic syndrome, and aging-related disorders, among others [22, 24, 28, 32, 38, 42, 43, 47, 49].

Referring to chronic liver disease, it has been intimately related to IGF-1 deficiency since the late 80s, and this idea has been consolidated over the following years. Decreased levels of free IGF-1 have been observed in patients with chronic liver disease, despite the normal or elevated GH secretion [12, 48]. A wide series of murine experimental studies in CCl_4_-induced cirrhosis showed that low doses of IGF-1 were able to (1) improve liver function, cholestasis, histopathology, and liver architecture, reducing oxidative damage [4, 21, 35]; (2) restore mitochondrial dysfunction, increasing mitochondrial membrane potential and ATP synthesis and diminishing intramitochondrial free radical production [4, 40]; (3) normalize intestinal absorption of sugars and amino acids in both compensated cirrhosis and cirrhosis with ascites, acting on enterocyte cytoskeleton [5, 9, 39]; and (4) improve osteopenia, increasing bone mass [13, 14], and improve testicular atrophy and steroidogenesis (all closely related alterations in hepatic disease), recovering testicular-blood-barrier integrity [6, 8, 11]. Additionally, one clinical trial in patients with cirrhosis of different etiologies demonstrated a significant increase of albumin serum levels when administered IGF-1 [17]. Furthermore, another murine study of CCl_4_-induced-cirrhosis suggested that IGF-1 treatment improves the polarity of hepatocytes and intercellular unions, leading to an improvement of liver architecture [45]. Bringing all these results together, it seems that the damaged liver could become a target organ for IGF-1 and thereby expressing the IGF-1 receptor. On the other hand, a specific and not well-understood IGF-1 activity might consist of contributing to cell polarity, acting on cytoskeleton [5, 11] and maintaining the normal hepatic architecture [4, 45].

In order to explore these possibilities, we conducted the present protocol using an experimental model of partial IGF-1-deficient mice [45] in which heterozygous *igf1* ^*+/−*^ were employed as null mice are not viable and because a partial IGF-1 deficiency resembles the human pathology. In this work, we examine liver histopathology and hepatic expression of genes encoding proteins of cytoskeleton, tight junctions, desmosomes, and extracellular matrix, as well as its regulators—gene-encoding metalloproteases (MMPs). Additionally, we extended our study by analyzing liver expression of genes encoding IGF-1, IGF-1R, and proteins involved in inflammatory and acute phase response.

## Materials and methods

### Animals and experimental design

The experimental model was established and characterized as previously reported by our group [10]. Briefly, IGF-1 heterozygous mice (Hz) were obtained by crossbreeding transgenic mice line 129SV^igf1tm1Arge^ and MF1 non-consanguineous strain [30].

Animal genotype determination was performed by PCR analysis (Applied Biosystems, 2720 Thermal Cycler, Spain). DNA was extracted from a piece of tail, and specific primers were used to identify both *igf-1* and *neo* genes (Extract-N-Amp TM Tissue PCR KIT Sigma, USA).

Animals were housed in cages inside a room with a 12-h light/dark cycle and constant humidity (50–55%) and temperature (20–22 °C). Food (Teklad Global 18% protein rodent diet, Harlan Laboratories, Spain) and water were given ad libitum. All experimental procedures were performed in compliance with the Guiding Principles for Research Involving Animals from the European Communities Council Directive of 24 November 1986 (86/609/EEC) and approved by the San Pablo-CEU University (Madrid) Bioethical Committee.

Three groups of 25 ± 2-week-old male mice were included in the experimental protocol: controls, wild-type mice (WT, *igf-1* ^+/+^, *n* = 10); untreated heterozygous mice (Hz, *igf-1* ^+/−^, *n* = 10); and heterozygous mice subcutaneously treated with low IGF-1 doses of 20 μg/kg/day for 10 days (Hz + IGF-1, *igf-1* ^+/−^, *n* = 10. We estimate this as a “low dose” compared to average doses commonly used which range from 80 to 4 mg/kg/day for weeks or months. Both WT and Hz groups received the administration vehicle in parallel, during the 10 days of treatment period. Chiron Corporation, USA, provided IGF-1.

On the 11th day, mice were weighed out and blood was extracted from the submandibular vein, and thereafter, the animals were sacrificed by cervical dislocation. The liver was carefully dissected out and divided into three sections: the left lobe was stored in RNAlater (Qiagen-Izasa, Spain) at −80 °C for microarray and PCR genetic analyses; the first half-right lobe was fixed in 4% paraformaldehyde for histological studies; and the second half-right lobe was placed in cryotubes and subsequently snap-frozen by submerging in liquid nitrogen and stored at −80 °C for posterior oxidative damage determinations.

### Serum IGF-1 concentrations and liver lipid peroxidation measurement

Serum IGF-1 levels were determined by ELISA method in a Varioskan spectrophotometer (Thermo Scientific, Spain) and interpreted using Skanlt® software, following specific commercial assay protocol instructions (Chiron Corporation, USA).

Malondialdehyde (MDA), widely used as an index of lipid peroxidation, was measured. N-methyl-2-phenylindole forms a stable chromophore with MDA at 45 °C for 60 min in 37% (12 N) hydrochloric acid medium. MDA from tissue homogenates was then quantified by colorimetric assay at 586 nm (Hitachi U2000 Spectro; Boehringer Mannheim) using the available commercial kit Oxis LPO-586 (Bioxytech; OXIS International Inc., Portland, OR, USA). MDA concentrations were then determined extrapolating against a standard curve. Determinations were performed in liver tissue homogenates embedded in Tris–HCl solution (1 g of liver tissue per 10 ml) centrifuged at 3000×*g* for 10 min at 4 °C.

### Histological analysis

Right liver lobe longitudinal sections were stained with H&E and Masson’s trichrome (4 μm thick, Reichert-Jung 2030 Biocut Microtome, Leica Microsystems, Germany). Tissue analyses and descriptions were made in three different areas from each sample double blinded by two different observers using a light microscope (Leica, Switzerland).

### Gene expression studies

#### Microarrays analysis

Liver mRNA was isolated from animals belonging to the three experimental groups in accordance with the protocol outlined in RNAqueousH-Micro Kit (Ambion, USA). Technical procedures for microarray analysis, including quality control of mRNA, labeling, hybridization, and scanning of the arrays, were performed according to standard operating procedures for Affymetrix protocols (GeneChipH Expression Analysis Manual, Affymetrix, USA). The mRNAs were profiled using Affymetrix HT MG-430. The array signals were normalized using Robust Multichip Averages [25], and batch effects of the three replicates were corrected using ComBat [26]. Differentially expressed genes between Hz vs. WT and Hz + IGF-1 vs. Hz samples were selected using FDR-corrected *p* value of 0.01 (*p* value of <0.05).

#### Total RNA extraction, reverse transcription, and RT-qPCR

The left hepatic lobe was included in RNAlater (Qiagen-Izasa, Spain). PCR assays were performed on samples of conserved tissue, which were homogenized with TRIzol reagent (Invitrogen, UK) by Tissue Lyser LT (Qiagen-Izasa, Spain), and RNA was extracted and purified using the RNeasy Mini Kit (Qiagen-Izasa, Spain) including digestion with RNase-free DNase, according to the manufacturer’s instructions. RNA quality was verified by the A260/A280 ratio and with the Bioanalyzer 2100 (Agilent Technologies Inc., USA). Purified RNA was then converted to cDNA by using the RNA-to-DNA EcoDryTM Premix (Clonetech Labs, USA) for q-PCR assays. Quantitative real-time PCR assays were performed in a 3100 Avant Genetic Analyzer (Applied Biosystems Hispania, Spain). The thermal profile consisted of an initial 5-min melting step at 95 °C followed by 40 cycles at 95 °C for 10s and 60 °C for 60s.

Specific Taqman® probes for the selected genes (*actb*, *aif1*, *c1qa*, *c1qb*, *cat*, *ccl6*, *ccr5*, *cdh1*, *cdh5*, *cldn1*, *cldn14*, *cldn7*, *csf1r*, *ctgf*, *dsc2*, *gadd45a*, *grb2*, *igf1*, *igf1r*, *il10rb*, *jam2*, *jun*, *fos*, *ly96*, *lyz2*, *mark2*, *myo1b*, *nras*, *pck1*, *pdk4*, *saa1*, *spna2*, *stk11*, *tubb2a*, *vcl*, *vim*) were supplied by Applied Biosytems.

The relative mRNA expression levels of the genes of interest were normalized to Tbp expression using the simplified comparative threshold cycle delta, cycle threshold (CT) method [2^−(ΔCT gene of interest−ΔCT actin)^] [31].

### Statistical analysis

All data represent mean ± SEM. Statistical analysis was performed on SPSS v22, (Statistical Package for the Social Sciences, USA). Significance was estimated by analysis of variance (ANOVA), followed by post hoc analysis. When appropriate, a confirmatory *t* test for comparison between means of specific variable pairs was undertaken. Correlation between IGF-1 and weight was analyzed by Pearson’s test. Differences were considered significant at a level of *p* < 0.05.

## Results

### Significant correlation between serum levels of IGF-1 and body weight

Circulating levels of IGF-1 were statistically lower in the untreated Hz group when compared to the WT group (Hz 534.1 ± 52.4 ng/mL and WT 696.1 ± 59.2 ng/mL, *p* < 0.01). Moreover, the Hz + IGF-1 group showed an increase of IGF-1 levels as compared to the Hz group (681.3 ± 7.7 ng/mL, *p* < 0.01), reaching similar values when compared to controls (WT). Therefore, low doses of IGF-1 therapy managed to normalize serum levels of IGF-1 in Hz mice.

With regard to body weight, a significant reduction was found in the untreated Hz group (Hz, 33.6 ± 0.4 g) compared to controls (WT, 40.1 ± 1.2 g, vs. Hz *p* < 0.001). However, treatment was able to bring up the weight to normal values as no difference was observed among treated Hz animals as compared to controls (Hz + IGF-1, 39.6 ± 0.8 g, vs. WT not significant; vs. Hz *p* < 0.01). A direct and significant correlation was observed between circulating IGF-1 levels and body weights (Pearson’s r 0.78, *p* < 0.001).

### Histological study

**Fig. 1:**
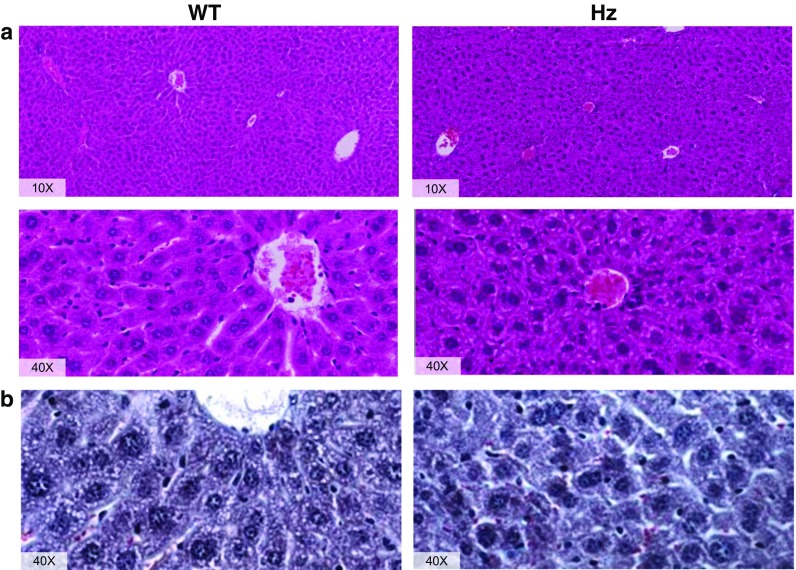
Histology: **a** H&E and **b** Masson’s trichrome staining of liver sections from both, WT (*left*) and Hz (*right*) animals

The conventional histological study of the liver tissue, stained with H&E, revealed an altered hepatic architecture in the Hz group. In contrast to WT animals, in which liver architecture and unions between hepatocytes are preserved (see Fig. 1a), Hz mice show clumps of cells because of defective unions between hepatocytes disturbing sinusoidal spaces (see Fig. 1a) and their relation with the centrilobular vein. Moreover, nuclei in the WT group seem to be properly stabilized and polarized while on the Hz group, some cells seem to be enucleated and most of nucleus appears aberrant, suggesting apoptotic cells. Also, abundant vacuolization can be seen in the Hz animals where the WT do not present any, reaffirming the idea of apoptotic cells. According to cell shape, when examining the WT group, it can be readily perceived a proper polyhedral shape compared to a more rounded shape of the Hz hepatocytes. All of this suggests an apparent disruption of cell polarity, which will be further discussed relating such alterations with the cytoskeletal disturbances found. Masson’s trichrome (Fig. 1b) confirms such misalignments, and although no centrilobular vein appears in the preparation, it can be found that red blood cells (stained in red) flow through sinusoidal spaces in WT animals, whereas in Hz, they do not follow any particular order. Masson’s staining also reveals no fibrosis, consistent with the fact that no external or chemical insult was applied to these animals.

### Liver gene expression studies

Microarray technology allowed us to identify about 120 genes which exhibit a fold change over ±1.5 in Hz as compared to WT mice, as well as Hz + IGF-1 compared to WT animals, all such genes being relevant to the purpose of this work.

#### Gene expression of IGF-1 and its receptor

**Fig. 2:**
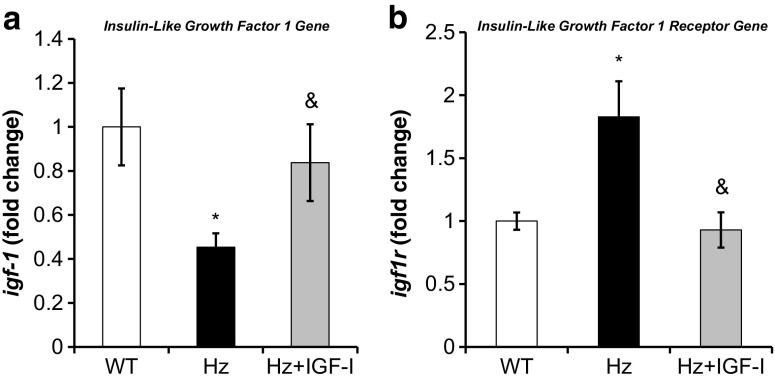
Real-Time qPCR analysis of **a** IGF-1 (*igf1*) and **b** IGF-1 receptor (*igf1r*) gene expression from liver homogenate in the three experimental groups. *p < 0.05 Hz group vs. controls (WT); ^&^ *p* < 0.05 IGF-1 treated Hz group (Hz + IGF-1) vs. untreated Hz group (Hz); *p* = ns Hz + IGF-1 vs. controls

As illustrated by Fig. 2a, a significant underexpression of *igf1* was found in Hz as compared to control group (WT). Low doses of IGF-1 were able to restore normal liver expression of the *igf1* gene, contributing to normalize circulating levels of this hormone.

**Fig. 3:**
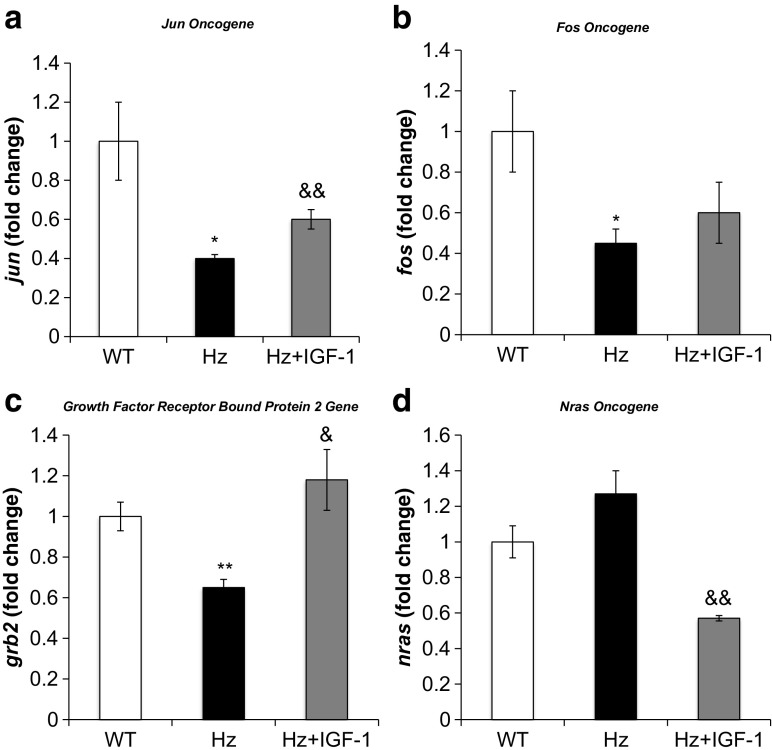
Real-time qPCR genetic expression analysis of intracellular signaling pathways of IGF-1 receptor: **a** Jun oncogene (*jun*), **b** FBJ osteosarcoma oncogene (*fos*), **c** growth factor regulator bound protein 2 (*grb2*), and **d** neuroblastoma Ras oncogene (*nras*) from liver homogenate in the three experimental groups. *p < 0.05, **p < 0.01 Hz group vs. controls (WT); ^&^ *p* < 0.05, ^&&^ *p* < 0.01 Hz + IGF-1 group vs. untreated Hz group (Hz); in all cases, *p* = ns between Hz + IGF-1 and control groups

Interestingly, *igf1r* was overexpressed in untreated Hz mice liver, and IGF-1 replacement therapy reduced *igf1r* expression to similar values to those found in the control group (WT) (Fig. 2b). This finding prompted us to study the expression of genes involved in IGF-1 intracellular signals (Table 1, Supplementary Table 1). RT-qPCR analysis confirmed that expression of *fos*, *jun*, and *grb2* genes was reduced in untreated Hz mice as compared to controls (WT group), whereas IGF-1 treatment normalized the expression of these genes (Fig. 3a–c). However, *nras* gene was found slightly increased, while IGF-1 therapy induced a significant reduction of its expression (Fig. 3d).

**Table 1:** Microarray significant results shown in fold change with *p* values

Protein	Gene	Hz vs WT (fold change)	*P* value	Hz + IGF-1 vs Hz (fold change)	*P* value
IGF-1R and Intracellular signaling					
Insulin-like growth factor 1 receptor	*igf1r*	1.44	0.0001	1.29	0.001
Neuroblastoma ras oncogene	*nras*	1.53	0.0004	−1.08	0.001
Jun oncogene	*jun*	−2.03	0.0006	1.05	0.01
FBJ osteosarcoma oncogene	*fos*	−1.44	0.001	−1.16	0.13
Inflammation and apoptosis					
Chemokine (C-C motif) receptor 5	*ccr5*	4.38	0.0001	−4.87	0.0001
Chemokine (C-C motif) ligand 6	*ccl6*	4.34	0.0002	−4.02	0.0001
Colony stimulating factor 1 receptor	*csf1r*	2.49	0.0004	−2.41	0.0006
Complement component 1, q subcomponent, α p	*c1qa*	5.40	0.0002	−3.43	0.0006
Lysozyme 2	*lyz2*	2.99	0.0019	−2.31	0.004
Lymphocyte antigen 96	*ly96*	1.71	0.003	−1.38	0.04
Interleukin 10 receptor beta	*il10rb*	1.75	0.004	−1.80	0.004
Serum amyloid A 1	*saa1*	3.74	0.0004	−1.70	0.006
Secreted phosphoprotein 1	*spp1*	2.44	0.001	−1.80	0.005
Allograft inflammatory factor 1	*aif1*	1.95	0.0001	−2.82	0.00006
Apoptotic peptidase activating factor 1	*apaf*	1.05	0.05	−1.05	0.05
Cytoskeleton					
Tubulin, beta 2A	*tubb2a*	−2.49	0.001	1.56	0.002
Vinculin	*vcl*	−1.49	0.003	1.31	0.06
MAP/microtubule affinity-regulating kinase 2	*mark2*	−1.52	0.01	1.34	0.05
Actin, beta	*actb*	1.65	0.0006	−1.58	0.0001
Serine/threonine kinase 11	*stk11*	−1.38	0.019	1.36	0.05
Myosin IB	*myo1b*	1.43	0.05	−1.37	0.03
Intercellular junctions					
Claudin 1	*cldn1*	−1.04	0.21	−1.23	0.18
Claudin 7	*cldn7*	1.48	0.003	−1.15	0.12
Claudin 14	*cldn14*	2.37	0.0006	−1.78	0.0003
Junction adhesion molecule 2	*Jam2*	1.23	0.03	−1.60	0.006
Cadherin 5	*cdh5*	1.70	0.0006	−1.80	0.0001
Desmocollin 2	*dsc2*	1.46	0.003	−1.46	0.001
Extracellular matrix					
Integrin beta 2	itgb2	−2.46	0.0006	2.03	0.0001
Connective tissue growth factor	ctgf/bp8	−1.60	0.001	−1.57	0.006
Proteoglycan 4	prg4	2.21	0.006	−2.24	0.0001
Collagen, type I, alpha 2	col1a2	−1.58	0.001	1.13	0.02
Collagen, type III, alpha 1	col3a1	−1.71	0.0003	1.08	0.38
Collagen, type V, alpha 2	col5a2	1.66	0.0001	−3.99	0.0006
Fibronectin 1	fn1	1.50	0.005	−1.45	0.003

Additionally, findings from the histopathological study may suggest some degree of inflammation in the IGF-1-deficient mice (Hz group).

#### Hepatic gene expression of inflammatory and acute phase response proteins

**Fig. 4:**
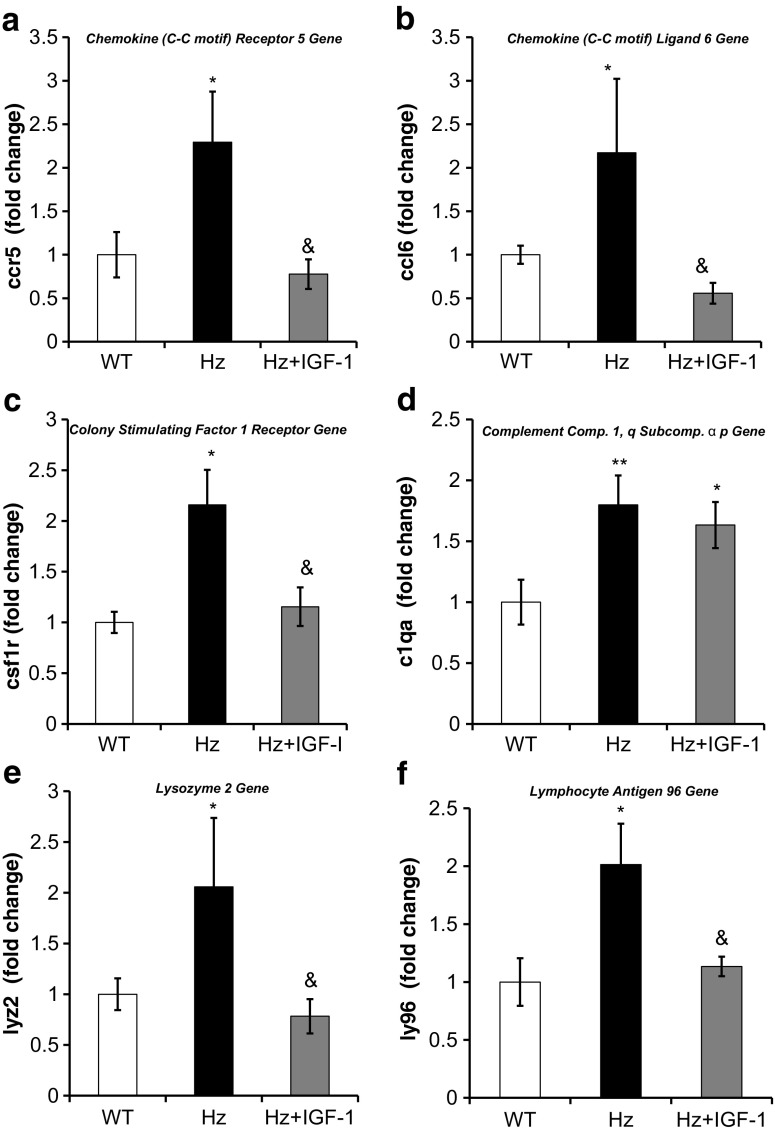
Real-time qPCR genetic expression analysis of proteins involved in inflammation: **a** chemokine (C-C motif) receptor 5 (*ccr5*); **b** chemokine (C-C motif) ligand 6 (*ccl6*); **c** colony stimulating factor 1 receptor (*csf1r*); **d** complement component 1, q subcomponent, alpha polypeptide (*c1qa*); **e** lysozyme 2 (*lyz2*); and **f** lymphocyte antigen 96 (*ly96*) from liver homogenate in the three experimental groups. *p < 0.05, **p < 0.01 untreated Hz vs. controls; ^&^ *p* < 0.05 Hz + IGF-1 group vs. untreated Hz group (Hz); in all cases, no statistical difference between Hz + IGF-1 and control groups

**Fig. 5:**
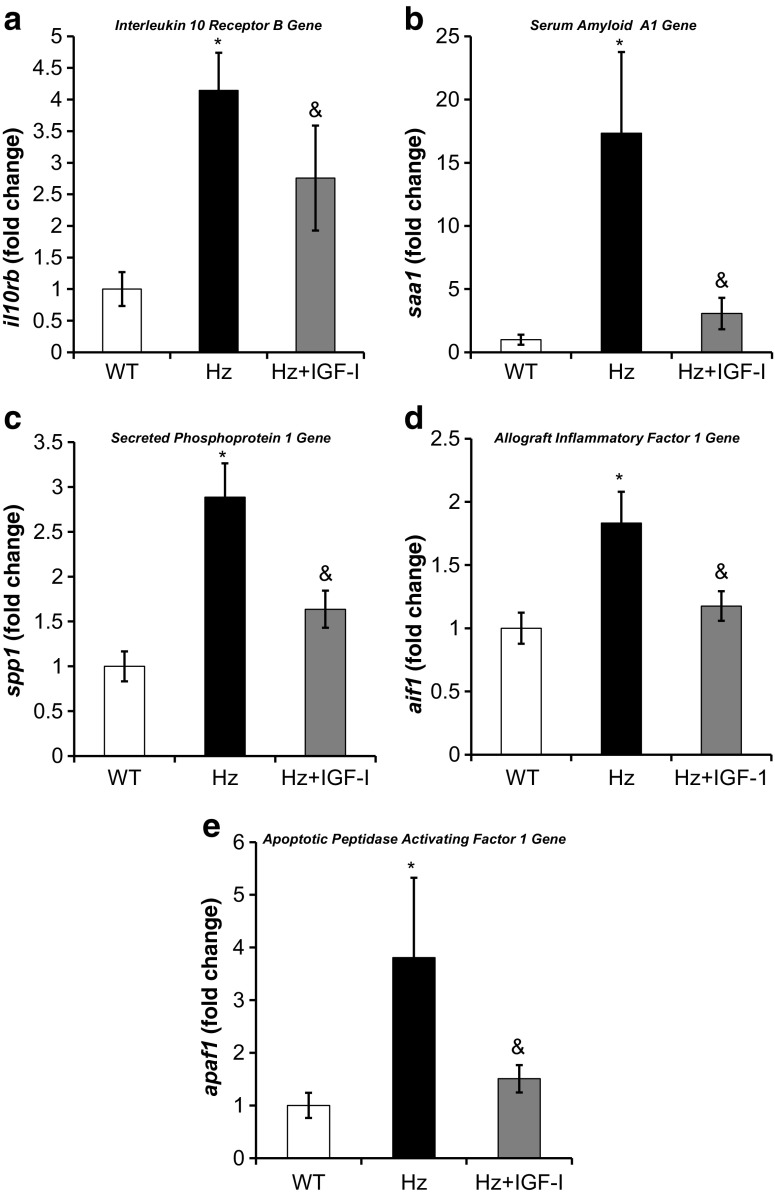
Real-time qPCR genetic expression analysis of proteins involved in inflammation and apoptosis: **a** interleukin 10 receptor B (*il10rb*), **b** serum amyloid A1 (*saa1*), **c** secreted phosphoprotein 1 (*spp1*), **d** allograft inflammatory factor 1 (*aif1*), and **e** apoptotic peptidase activating factor 1 (*apaf1*) from liver homogenate in the three experimental groups. *p < 0.05, **p < 0.01 untreated Hz vs. controls; ^&^ *p* < 0.05 Hz + IGF-1 group vs. untreated Hz group (Hz); in all cases, no statistical difference between Hz + IGF-1 and control groups

Despite that none of the groups received inflammatory or oxidative injury, the Hz group showed an overexpression of several genes that encode proteins related to inflammatory and acute phase response (Table 1, Supplementary Table 2), confirmed by qRT-PCR. A significant increase in the expression of the genes *c1qa*, *ccr5*, *csf1r*, *il10rb*, *ly96*, *saa1*, and *spp1* was found in untreated Hz mice as compared to controls (WT group), while no differences were found in the expressions of *c1qb*, *ccl6*, and *lyz2*. Moreover, the Hz + IGF-1 group displayed a normalized gene expression of *ccr5*, *ccl6*, *il10rb*, *csf1r*, *saa1*, *spp1*, *lyz2*, and *ly96* (Figs. 4 and 5a–c). No change in genes related to the complement proteins (*c1aq* and *c1qb*) was observed in Hz + IGF-1 animals.

In addition, a significant overexpression of *aif1* and *apaf1* genes was found in untreated deficient mice (Hz group) as compared to both controls (WT group) and Hz + IGF-1 groups (Fig. 5d, e).

Complementarily, microarray studies also revealed a relevant overexpression of genes encoding acute-phase proteins (such as macrophage activation 2 like, orosomucoid 1 and 2, calgranulin A and B, etc.) as well as HLA class II genes. These findings deserve further studies for better understanding of their impact role in IGF-1 deficiency within the liver.

#### Liver expression of genes encoding for cytoskeleton and tight and adherent junctions and desmosomes

**Fig. 6:**
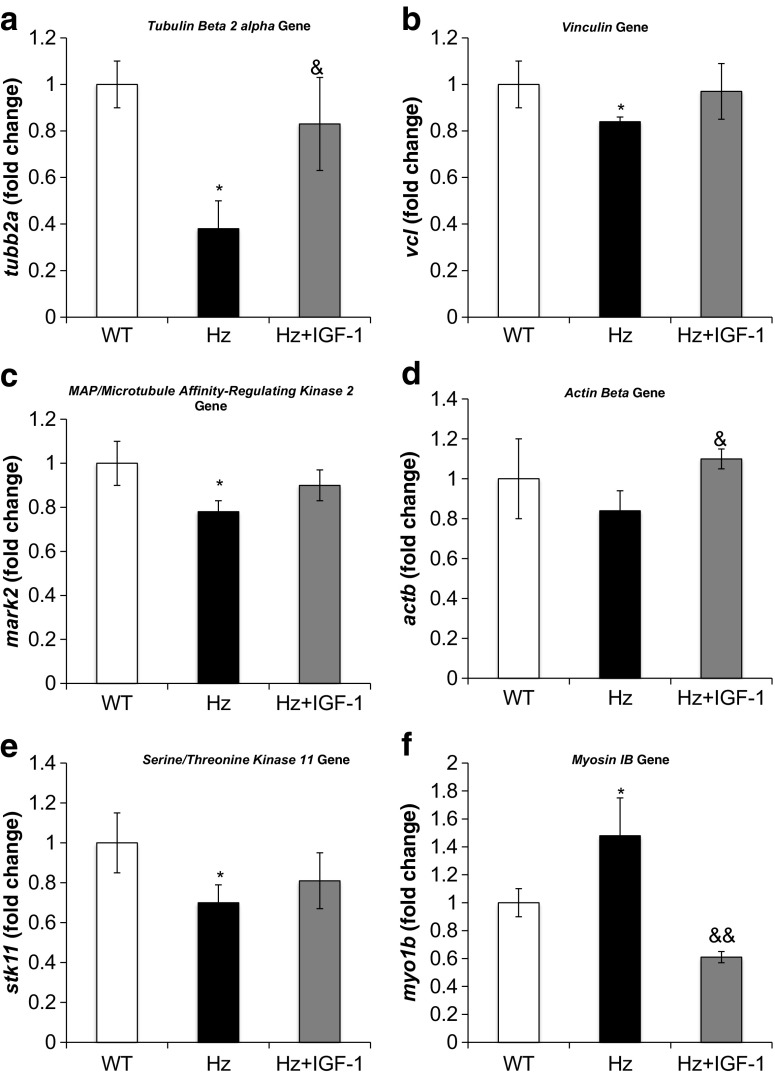
Real-time qPCR genetic expression analysis of proteins involved in the cytoskeleton: **a** tubulin beta 2 Alpha (*tubb2a*), **b** vinculin (*vcl*), **c** MAP/microtubule affinity-regulating kinase 2 (*mark2*), **d** actin beta (*actb*), **e** serine/threonine kinase 11 (*stk11*), and **f** myosin 1 beta (*myo1b*) from liver homogenate in the three experimental groups. *p < 0.05, **p < 0.01 Hz group vs. controls (WT); ^&^ *p* < 0.05, ^&&^ *p* < 0.01 Hz + IGF-1 group vs. untreated Hz group (Hz); in all cases, no statistical difference between Hz + IGF-1 and control groups

Table 1 (Supplementary Table 3) summarizes the microarray results, showing the expression of genes encoding cytoskeletal proteins. Some of these genes were selected for RT-qPCR analysis (*actb*, *mark2*, *myo1b*, *spna2*, *stk11*, *tubb2a*, *vcl*, and *vim*). It was found that IGF-1-deficient mice (Hz group) exhibited a reduction of liver-expressed genes coding for tubulin β2a, actin β, MAP-microtubule affinity-regulating kinase 2, vinculin, and espectrin α2 and a significant increase in myosin IB and vimentin genes. All the above mentioned genes were normalized in the Hz + IGF-1 group (Fig. 6), with the only exception of *spna2*, where IGF-1 treatment did not modulate at all the expression of this gene.

**Fig. 7:**
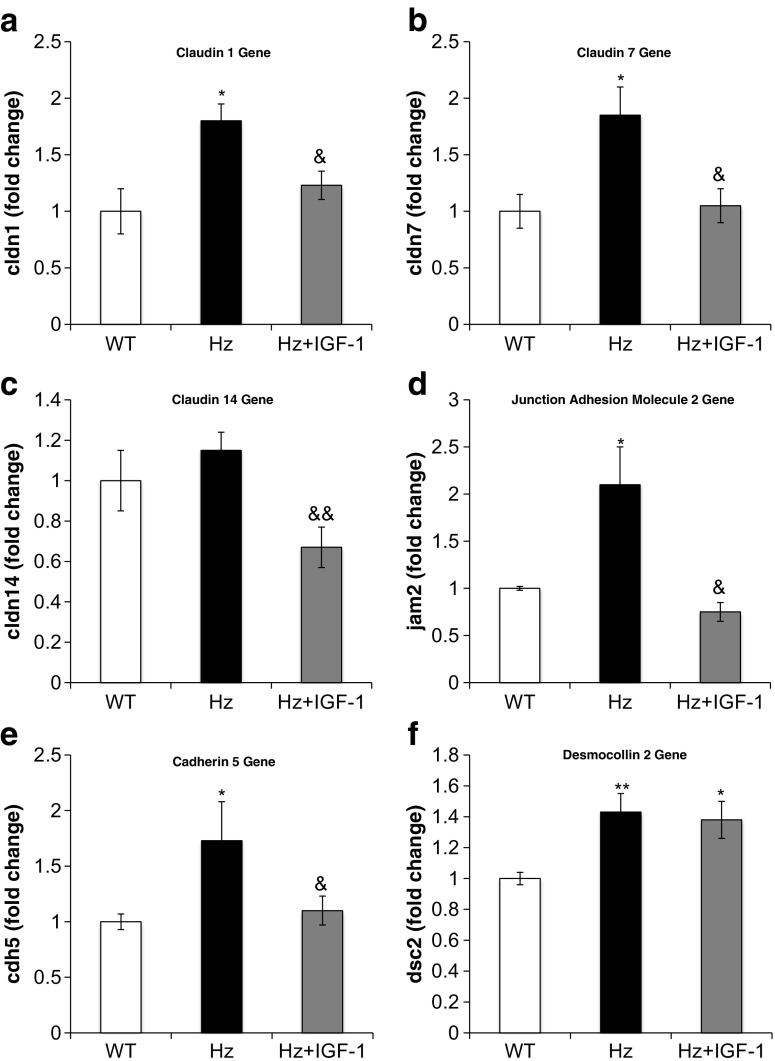
Real-time qPCR genetic expression analysis of proteins implicated in intercellular junctions: **a** claudin 1 (*cldn1*), **b** claudin 7 (*cldn7*), **c** claudin 14 (*cldn14*), **d** junction adhesion molecule 2 (*jam2*), **e** cadherin 5 (*cdh5*), and **f** desmocollin 2 (*dsc2*) from liver homogenate in the three experimental groups. *p < 0.05, **p < 0.01 Hz group vs. controls (WT); ^&^ *p* < 0.05, ^&&^ *p* < 0.01 Hz + IGF-1 group vs. untreated Hz group (Hz); in all cases, no statistical difference between Hz + IGF-1 and control groups

On the other hand, Table 1 (Supplementary Table 4) summarizes microarray findings regarding genes encoding for tight, adherent, and gap junctions and desmosomes. RT-qPCR analysis confirmed a significant overexpression as compared to WT group of genes encoding claudins 1, 7, and 14; cadherin 5; desmocollin 2; and jam 2. All of such were normalized by IGF-1 therapy (Hz + IGF-1 group) (Fig. 7a–e), with the only exception of *desmocollin 2* gene (Fig. 7f).

#### Hepatic expression of genes related to proteins of extracellular matrix and growth factors involved in ECM establishment

**Fig. 8:**
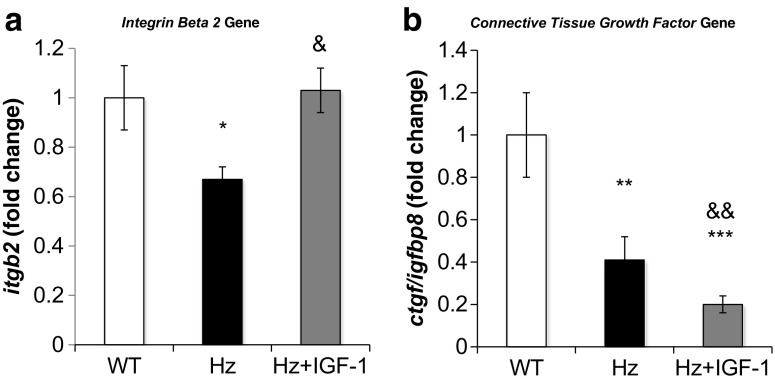
Real-time qPCR genetic expression analysis of proteins implicated in extracellular matrix: **a** integrin beta 2 (*itgb2*) and **b** connective tissue growth factor (*ctgf/igfbp8*) from liver homogenate in the three experimental groups. *p < 0.05, **p < 0.01, ***p < 0.001 Hz group vs. controls (WT); ^&^ *p* < 0.05, ^&&^ *p* < 0.01 Hz + IGF-1 group vs. untreated Hz group (Hz); in all cases, no statistical difference between Hz + IGF-1 and control groups

Microarray analysis showed an abnormal underexpression or overexpression of genes encoding extracellular matrix (ECM) or growth factors involved in the ECM establishment (Fig. 8a). Collagen 1a2, collagen 3a1, integrin β2, and connective tissue growth factor (*ctgf/igfbp8*) genes were found underexpressed in deficient IGF-1 mice (Hz group), whereas genes for collagen 5a2, fibronectin 1, proteoglycan 4, or hyaluronan 2 seemed to be overexpressed. Interestingly, all these genes from the Hz + IGF-1 group showed values similar to those found in controls (WT group), with the only exception of connective tissue growth factor (Fig. 8b, c).

#### Hepatic oxidative damage

In order to gain more insight into the mechanism involved in the histological damage found in IGF-1-deficient mice, hepatic levels of MDA, as a marker of lipid peroxidation, were determined. It was observed that untreated IGF-1-deficient mice (Hz group) had significantly increased levels of hepatic MDA (5.28 ± 1.04 μM) as compared to controls (WT group; 1.75 ± 0.54 μM; *p* < 0.05), whereas IGF-1 replacement therapy was able to significantly reduce liver oxidative damage (Hz + IGF-1; 0.96 ± 0.55 μM; *p* < 0.05 vs. Hz and not significant vs. WT group).

## Discussion

Results in this paper clearly show that the mere IGF-1 partial deficiency is associated with relevant alterations of the hepatic architecture, paired with an altered expression pattern of genes encoding cytoskeleton proteins, as well as genes related to hepatocyte polarity, cell junctions, and extracellular matrix proteins. These findings strongly suggest that IGF-1 deficiency results in profound modifications of gene expression patterns.

In addition, the single IGF-1 partial deficiency—without exogenous insults—induced the hepatic expression of the gene coding for IGF-1 receptor and a remarkable response of acute-phase proteins, inflammation, and hepatic oxidative damage. However, further studies on IGF-1R translocation to the membrane and degradation rates should be assessed to conclude its functionality and availability.

### Experimental model of IGF-1 deficiency and dose selected for replacement therapy

The present work falls within a series of studies using an experimental model of haploinsufficiency, in which mice hypoexpressing *igf1* gene (*igf1* ^*+/−*^, Hz; heterozygous group) show low circulating IGF-1 levels [10, 11, 24]. The preference for IGF-1 treatment at low doses was chosen following the experience acquired from previous studies belonging to the same model [4–6, 8, 9, 11, 13, 14, 18, 21, 24, 35, 39, 40, 45]. Briefly, such dosage is enough to restore normal circulating IGF-1 values without inducing hypoglycemia and any adverse effects.

As previously reported in this experimental model [10, 11, 18, 24], the body weight of IGF-1-deficient mice was significantly lower than of their wild-type counterparts, and substitutive treatment corrected such deficiency. However, although absolute liver weight also exhibited differences in the same sense, and liver weight relative to total body weight was not different among experimental groups. Therefore, the liver was proportional to the animal size. These findings sound coherent since IGF-1 is the executioner growth factor of GH, being the most powerful anabolic hormone in mammals.

### Relevant response of acute-phase and inflammation proteins resulting to oxidative damage and histopathology

A surprising response of acute-phase proteins (macrophage activation 2 like, orosomucoid 1 and 2, calgranulin 1 and 2, etc.), as well as genes encoding HLA-class II antigens, involved in the antigen-presentation, starting the cellular and humoral immunological response, resulting in inflammation. This finding requires further procedures in order to gain more insight into the impact of IGF-1 deficiency on such hepatic routes [44].

Among the genes associated with inflammation, the *aif1* and *apaf1* genes were included in this study, which code for allograft inflammatory factor 1 and apoptotic peptidase activator factor 1 [20]. Although apoptotic pathways were included in this study, these factors deserve specific attention. APAF-1, a well-recognized player of the apoptotic pathway, which underlies the activation of caspase-9, also seems to be involved in cell differentiation and the regulation of cell cycle in response to DNA-damaging agents, while AIF, a caspase-independent cell death effector, is involved in the control of adipocyte differentiation and mitochondrial metabolism regulation [20]. Surprisingly, both of these proteins showed definitive overexpression in untreated Hz mice, and substitutive treatment reversed such expression to normal values. This could mechanistically indicate that intrinsic apoptotic pathways were overreacting, even with no harm to the animal. Such finding could be explained by altered hepatic architecture and concomitant cessation of survival signals that adhesion molecules transmit when appropriately attached (anoikis).

Another result that deserves special mention is the altered expression of genes encoding cytoskeletal proteins. In hepatocytes, epithelial cell differentiation generates basal lamina membrane at cell-cell contact sites (bile canaliculi) orientating microtubules to aim the proper polarization of the cell [16].

Untreated Hz mice showed a significant reduction of two genes implicated in microtubule structure and stability: tubulin β 2A and MAP/microtubule affinity-regulating kinase 2. Of interest, treatment was effective as no differences were found between controls and the IGF-1 treated Hz group (Fig. 6a, c). Such finding might explain the aberrant histological observations in untreated Hz mice, which suggested an altered hepatocyte polarity.

### The mere IGF-1 deficiency makes the liver a target organ for IGF-1

One of the major findings of this paper is the association between the partial IGF-1 deficiency and the significant expression of IGF-1, the receptor in the liver, suggesting that IGF-1 contributes to the liver homeostasis. The lack of expression of the IGF-1 receptor in animals that have not received any injury seems to mean that it is a “defense mechanism,” as previous results suggested [4, 21, 35, 40, 41, 45]. Such defense mechanism allows the liver to take advantage of the beneficial actions of this hormone following injury. In spite, this study shows how mere IGF-1 deficiency acts as “harmful agent” altering cytoskeletal components and normal ECM establishment, modifying expression of genes showing a genetic pattern of inflammation and oxidative stress with a relevant response of acute-phase proteins.

A direct effect of IGF-1 on the liver is an astonishing novel concept in hepatology, since until now, it has been recognized that the liver is not a target organ for IGF-1, supported by the absence of its receptor in liver cells under physiological conditions [3, 33, 34, 50]. However, in the last decades, accumulated evidence have suggested that a damaged liver must be expressing the IGF-1 receptor since short cycles of low doses of IGF-1 induced many beneficial effects on the hepatic parenchyma and function [4, 17, 21, 35, 40, 45].

The autocrine regulation of IGF-1 was first demonstrated by Caro et al. in 1988 [3]. The diverse activities of IGF-1 in stimulating mitogenesis, increasing substrate uptake and metabolic activity, inhibiting apoptosis, and modulating a variety of specific functions in highly differentiated cell types are, in most part, mediated through binding and activation of the type I IGF1 receptor. Generally, the interaction of an IGFBP with IGF-1 blocks the receptor activation and thus its effects at all levels [1, 36]. Perhaps, this could be one of the mechanisms behind our findings regarding overexpression of *igf1r* and hypoexpression of *igfbp1*, *igfbp2*, and *igfbp5* [18] and *igfbp8*. In turn, this decrease in function could also explain the observed changes in liver cellular architecture, the proinflammatory profile found, and even the intracellular derangements observed.

On the other hand, overexpressed *igf1r*, which under physiological conditions is virtually absent in hepatic parenchymal cells in adult rats, mice, and humans, induced important changes in intracellular signaling pathways [3, 34].

Under normal conditions, once IGF-1 binds to its receptor and activates intracellular signaling, IRS-1 with the concourse of PI3K, Shp2, Grb2, and Sos activate Shc, which in turn activates Ras. The Ras pathway is a prominent signaling pathway for cell proliferation and survival [19].

IGF-1 deficiency in our Hz mouse model resulted in overexpression of igf1r and nras and downregulation of grb2 by unknown mechanism(s). We may speculate that the sole overexpression of *igf1r* could have triggered an aberrant activation of *nras*. Under normal conditions, *nras* and its analogs *hras*, *rras*, and *kras* activate the MAPK pathway, which in turn, together with *erk*, exerts intranuclear effects on *elk-1*, which aided with *ap-1* activates *cfos* and *cjun*. All these genes downstream regulate the expression of genes involved in cell proliferation and survival and are key participants in anoikis, a special form of apoptosis triggered by the loss of cell-cell or cell-matrix anchorage [23].

Perhaps as a result of *nras* overexpression, *mapk1* was also overregulated in microarray studies, although below the established threshold to include it in the RT-qPCR panel. Unexpectedly, *fos* and *jun* were underregulated, probably as an autoprotective mechanism developed by the liver cells to avoid death. It would be helpful to further explore *elk-1* and *ap-1*. Interestingly, substitutive treatment normalized the expression of *igf1*, *igf1r*, *grb2*, and *nras*, while no differences in *fos* or *jun* were observed (Fig. 3a, b).

In this sense, the reduction of *igfbp8* may deserve a particular mention because it has included a domain for connective tissue growth factor (CTGF). This molecule is a multimodular protein that resides in the extracellular matrix, which binds and coordinates biologically active factors involved in ECM establishment under physiological conditions [11, 37]. CTGF has its own IGF-1 recognition domain (thus acting also as IGFBP-8), suggesting a role for IGF-1 deficiency in TGFβ activation (a well-known collagen production stimulator) [46]. In this study, the partial IGF-1 deficiency was associated to *ctgf* (or *igfbp8*) gene underexpression and thus suggesting an altered ECM establishment, in accordance with previous findings on testicle using this same experimental model [11]. Although IGF-1 therapy did not increase *ctgf* expression in the liver, it did show effectiveness on testes [11].

## Conclusions

In conclusion, partial IGF-1 deficiency leads to liver expression of genes encoding IGF-1 receptor and numerous proteins involved in acute-phase and inflammation responses, resulting in liver oxidative damage. This response resembles that of a pathological liver, which typically shows altered expression of genes related to cytoskeletal proteins, hepatocyte polarity, cell junctions, and extracellular matrix proteins as occurs in these animal’s livers.

## Electronic supplementary material

Supplementary table 1 (DOCX 35 kb)

Supplementary table 2 (DOCX 101 kb)

Supplementary table 3 (DOCX 94 kb)

Supplementary table 4 (DOCX 105 kb)

## Abbreviations

*ANOVA*: Analysis of variance
*CCl*_*4*_: Tetrachloride
*CT*: cycle threshold
*ECM*: Extracellular matrix
*ELISA*: Enzyme-linked immunosorbent assay
*GH*: Growth hormone
*HCl*: Hydrochloric acid
*H&E*: Hematoxylin and Eosin
*HLA*: Human leucocyte antigen
*Hz*: Heterozygous (*igf-1* ^*+/−*^)
*IGFBPs*: IGF-1 binding proteins
*IGF-1*: Insulin-like growth factor-1
*IGF-1R*: IGF-1 receptor
*IUGR*: intrauterine growth restriction
*MDA*: Malondialdehyde
*MMPs*: Metalloproteinases
*mRNA*: Messenger ribonucleic acid
*PBS*: Phosphate buffer saline
*PCR*: Polymerase chain reaction
RT-qPCR: Real-time quantitative polymerase chain reaction
*SEM*: Standard error of mean
*SPSS*: Statistical Package for the Social Sciences
*TGFβ*: Transforming growth factor β
*WT*: Wild-type, control group
