# Supplementary material for: IGF-1 modulates gene expression of proteins involved in inflammation, cytoskeleton, and liver architecture
Source: J Physiol Biochem. 2017 Jan 26;73(2):245–58. doi: 10.1007/s13105-016-0545-x (PMC5399066; doi:10.1007/s13105-016-0545-x)
Supplement: Supplementary file 1 — (DOCX 35 kb) [file 13105_2016_545_MOESM1_ESM.docx]

**Supplementary table 1.** IGF1R and intracellular signaling microarray liver gene expression.

| **Protein** | **Gene** | **Hz vs WT**  **(Fold change)** | **P Value** | **Hz+IGF-1 vs Hz**  **(Fold change)** | **P Value** | |
| --- | --- | --- | --- | --- | --- | --- |
| Insulin-like growth factor 1 receptor | *igf1r* | 1.44 | 0.0001 | 1.29 | | 0.001 |
| Insulin receptor | *insr* | -1.07 | 0.32 | 1.31 | | 0.08 |
| Insulin receptor substrate 1 | *irs1* | -1.17 | 0.27 | 1.17 | | 0.26 |
| Insulin receptor substrate 2 | *irs2* | -1.22 | 0.25 | 1.12 | | 0.15 |
| Insulin receptor substrate 3 | *irs3* | -1.12 | 0.15 | 1.12 | | 0.27 |
| Insulin receptor substrate 4 | *irs4* | -1.01 | 0.41 | 1.13 | | 0.20 |
| Phosphatidylinositol 3-kinase, catalytic, alpha polypeptide | *pik3ca* | -1.08 | 0.39 | 1.28 | | 0.15 |
| Phosphatidylinositol 3-kinase | *pik3r1* | -1.20 | 0.27 | 1.42 | | 0.07 |
| Phosphatidylinositol 3-kinase, catalytic, gamma polypeptide | *pik3cg* | -1.35 | 0.01 | 1.42 | | 0.005 |
| Phosphatidylinositol 3-kinase (p85 beta) | *pik3r2* | -1.43 | 0.06 | 1.29 | | 0.03 |
| Thymoma viral proto-oncogene 1 | *akt1* | 1.08 | 0.38 | -1.09 | | 0.33 |
| Thymoma viral proto-oncogene 2 | *akt2* | -1.08 | 0.35 | -1.11 | | 0.21 |
| Thymoma viral proto-oncogene 3 | *akt3* | -1.36 | 0.06 | 1.21 | | 0.09 |
| Growth factor receptor bound protein 2 | *grb2* | 1.54 | 0.0001 | -1.17 | | 0.002 |
| Son of sevenless homolog 1 (Drosophila) | *sos1* | 1.08 | 0.45 | -1.38 | | 0.006 |
| Harvey rat sarcoma virus oncogene 1 | *hras1* | 1.30 | 0.09 | 1.13 | | 0.15 |
| s-Ki-ras2 kirstenrat sarcoma viral oncogene homolog | *kras* | 1.00 | 0.57 | 1.00 | | 0.60 |
| Neuroblastoma ras oncogene | *nras* | 1.53 | 0.0004 | -1.08 | | 0.001 |
| V-raf-leukemia viral oncogene 1 | *raf1* | -1.26 | 0.01 | 1.09 | | 1.30 |
| Mitogen-activated protein kinase 1 | *map2k1* | 1.21 | 0.27 | -1.33 | | 0.007 |
| Mitogen-activatedproteinkinase 3 | *mapk3* | 1.01 | 0.45 | -1.01 | | 0.48 |
| Mitogen-activatedproteinkinase 1 | *mapk1* | 1.41 | 0.006 | -1.23 | | 0.007 |
| Mitogen-activatedproteinkinase 8 | *mapk8* | 1.09 | 0.38 | -1.25 | | 0.08 |
| Jun oncogene | *jun* | -2.03 | 0.0006 | 1.05 | | 0.01 |
| FBJ osteosarcoma oncogene | *fos* | -1.44 | 0.001 | -1.16 | | 0.13 |
| Cyclin D2 | *ccnd2* | -1.16 | 0.29 | 1.04 | | 0.35 |
| Cyclin B1 | *ccnb1* | 1.00 | 0.48 | -1.18 | | 0.39 |
| Cyclin D1 | *ccnd1* | -1.19 | 0.25 | -1.12 | | 0.31 |
| Cyclin E1 | *ccne1* | 1.96 | 0.0006 | -1.57 | | 0.001 |
| Cyclin-dependentkinase 2 | *cdk2* | 1.31 | 0.08 | -1.34 | | 0.07 |
| Cyclin-dependentkinase 4 | *cdk4* | 1.02 | 0.35 | 1.01 | | 0.41 |
| Cyclin-dependentkinase 6 | *cdk6* | 1.21 | 0.27 | -1.39 | | 0.08 |
| Cyclin-dependent kinase inhibitor 1A (P21) | *cdkn1a* | -1.52 | 0.003 | 1.22 | | 0.088 |
| Cyclin-dependent kinase inhibitor 1B | *cdkn1b* | 1.08 | 0.29 | -1.05 | | 0.32 |
| Cyclin-dependent kinase inhibitor 2A | *cdkn2a* | 1.22 | 0.37 | -1.16 | | 0.39 |
| E2F transcription factor 1 | *e2f1* | -1.04 | 0.49 | 1.14 | | 0.39 |
| Retinoblastoma 1 | *rb1* | 1.17 | 0.20 | -1.31 | | 0.07 |
| Forkhead box O1 | *foxo1* | -1.23 | 0.12 | 1.18 | | 0.33 |
| Mechanistic target of rapamycin (serine/threonine kinase) | *mtor* | 1.15 | 0.32 | 1.15 | | 0.25 |
| NFk light polypeptide gene enhancer in B-cells 1 | *nfkb1* | 1.09 | 0.35 | -1.19 | | 0.23 |
| NFk light polypeptide gene enhancer in B-cells 2 | *nfkb2* | -1.19 | 0.26 | 1.31 | | 0.06 |
| Proliferatingcell nuclear antigen | *pcna* | 1.35 | 0.10 | -1.35 | | 0.04 |

**Supplementary table 2.** Liver expression of genes encoding proteins involved in inflammatory and acute-phase response proteins.

| **Protein** | ***Gene*** | **Hz vs WT**  **(Fold change)** | **P Value** | **Hz+IGF-1 vs Hz**  **(Fold change)** | **P Value** |
| --- | --- | --- | --- | --- | --- |
| Apoptotic peptidase activating factor 1 | *apaf1* | 1.05 | 0.05 | -1.05 | 0.05 |
| Allograft inflammatory factor 1 | *aif1* | 1.95 | 0.0001 | -2.82 | 0.00006 |
| Colony stimulating factor 1 (macrophage) | *csf1* | 1.09 | 0.46 | 1.29 | 0.16 |
| Colony stimulating factor 1 receptor | *csf1r* | 2.49 | 0.0004 | -2.41 | 0.0006 |
| Colony stimulating factor 3 receptor (granulocyte) | *csf3r* | 1.04 | 0.37 | 1.08 | 0.41 |
| Colony stimulating factor 3 (granulocyte) | *csf3* | 1.18 | 0.12 | 1.02 | 0.52 |
| Colony stimulating factor 2 | *csf2* | -1.03 | 0.11 | 1.16 | 0.19 |
| Colony stimulating factor 2 receptor alpha | *csf2ra* | 1.35 | 0.018 | -1.30 | 0.019 |
| Colony stimulating factor 2 receptor beta 2 | *csf2rb2* | 2.38 | 0.0001 | -2.39 | 0.0006 |
| Interferon gamma receptor 1 | *ifngr1* | 1.62 | 0.0003 | -1.34 | 0.06 |
| Chemokine (C-C motif) ligand 11 | *ccl11* | 1.12 | 0.07 | -1.01 | 0.47 |
| Chemokine (C-C motif) receptor 6 | *ccr6* | -1.10 | 0.18 | 1.00 | 0.5 |
| Chemokine (C-C motif) ligand 6 | *ccl6* | 4.34 | 0.0002 | -4.02 | 0.0001 |
| Chemokine (C-C motif) receptor 5 | *ccr5* | 4.38 | 0.0001 | -4.87 | 0.0001 |
| Chemokine (C-C motif) ligand 12 | *ccl12* | 1.10 | 0.13 | -1.02 | 0.48 |
| Chemokine (C-C motif) ligand 5 | *ccl5* | 1.40 | 0.017 | -1.06 | 0.33 |
| Chemokine (C-C motif) ligand 17 | *ccl17* | 1.26 | 0.17 | -1.20 | 0.19 |
| Chemokine (C-C motif) ligand 3 | *ccl3* | -1.28 | 0.133 | -1.12 | 0.37 |
| Chemokine (C-C motif) ligand 4 | *ccl4* | 1.06 | 0.19 | 1.07 | 0.35 |
| Chemokine (C-X-C motif) ligand 1 | *cxcl1* | 1.66 | 0.0006 | -2.13 | 0.002 |
| Chemokine (C-X-C motif) ligand 14 | *cxcl14* | 4.06 | 0.00001 | -1.11 | 0.21 |
| Chemokine (C-X-C motif) ligand 16 | *cxcl16* | 1.43 | 0.03 | -1.58 | 0.03 |
| Interferon alpha 2 | *ifna2* | 1.06 | 0.11 | -1.05 | 0.28 |
| Interleukin 12a | *il12a* | 1.16 | 0.35 | 1.02 | 0.33 |
| Interleukin 12b | *il12b* | 1.23 | 0.019 | 1.09 | 0.20 |
| Interleukin 13 | *il13* | 1.06 | 0.38 | 1.11 | 0.15 |
| Interleukin 13 receptor alpha 1 | *il13ra1* | 1.43 | 0.01 | -1.61 | 0.005 |
| Leukemia inhibitory factor | *lif* | 1.03 | 0.42 | 1.06 | 0.42 |
| Oncostatin M receptor | *osmr* | 1.54 | 0.005 | -1.56 | 0.03 |
| Oncostatin M | *osm* | 1.15 | 0.42 | 1.00 | 0.28 |
| Secreted phosphoprotein 1 | *spp1* | 2.44 | 0.001 | -1.80 | 0.005 |
| Lysozyme 1 | *lyz1* | 3.26 | 0.0002 | -2.79 | 0.0017 |
| Lysozyme 2 | *lyz2* | 2.99 | 0.0019 | -2.31 | 0.004 |
| Solute carrier family 11 | *slc11a1* | 1.45 | 0.018 | -1.50 | 0.025 |
| Toll-like receptor 1 | *tlr1* | 1.52 | 0.01 | -1.40 | 0.28 |
| Lymphocyte antigen 96 | *ly96* | 1.71 | 0.003 | -1.38 | 0.04 |
| Complement component 3 | *c3* | 1.89 | 0.002 | -1.57 | 0.002 |
| Complement component 1, q subcomponent, α p | *c1qa* | 5.40 | 0.0002 | -3.43 | 0.0006 |
| Complement component 1, q subcomponent, ß p | *c1qb* | 6.61 | 0.0001 | -2.43 | 0.001 |
| Complement component 1, q subcomponent, C | *c1qc* | 3.32 | 0.0006 | -2.69 | 0.0006 |
| Macrophage activation 2 like | *mpa2l* | 3.11 | 0.0006 | -2.62 | 0.0001 |
| Orosomucoid 1 | *orm1* | 3.51 | 0.0017 | -2.44 | 0.0002 |
| Orosomucoid 2 | *orm2* | 10.52 | 0.0001 | -6.49 | 0.0001 |
| S100 calcium binding protein A8 (calgranulin A) | *s100a8* | 2.89 | 0.002 | 1.16 | 0.4 |
| S100 calcium binding protein A9 (calgranulin B) | *s100a9* | 2.00 | 0.002 | 1.20 | 0.14 |
| Serum amyloid A 1 | *saa1* | 3.74 | 0.0004 | -1.70 | 0.006 |
| Serum amyloid A 2 | *saa2* | 4.52 | 0.0004 | -2.62 | 0.0002 |
| Serum amyloid A 3 | *saa3* | 1.72 | 0.012 | -1.31 | 0.02 |
| Serum amyloid A 4 | *saa4* | 1.29 | 0.15 | -1.24 | 0.12 |
| Histocompatibility 2, class II antigen A, alpha | *h2-aa* | 6.56 | 0.0001 | -4.22 | 0.001 |
| Histocompatibility 2, class II antigen A, beta 1 | *h2-ab1* | 2.40 | 0.001 | -4.22 | 0.002 |
| Histocompatibility 2, class II antigen E beta | *h2-eb1* | 3.17 | 0.0002 | -2.97 | 0.001 |
| Arginase, liver | *arg1* | 1.33 | 0.13 | -1.37 | 0.08 |
| Eosinophilperoxidase | *epx* | -1.01 | 0.47 | 1.35 | 0.10 |
| Lactoperoxidase | *lpo* | -1.17 | 0.33 | 1.16 | 0.19 |
| Monoamine oxidase A | *maoa* | 1.06 | 0.37 | -1.20 | 0.21 |
| Monoamine oxidase B | *maob* | 1.11 | 0.33 | -1.24 | 0.09 |
| Peroxidasin homolog (Drosophila) | *pxdn* | 1.03 | 0.11 | -1.05 | 0.12 |
| Xanthine dehydrogenase | *xdh* | 1.42 | 0.03 | -1.34 | 0.14 |
| Interferon gamma | *ifng* | -1.13 | 0.34 | 1.12 | 0.38 |
| Interleukin 1 alpha | *il1a* | -1.01 | 0.54 | 1.04 | 0.57 |
| Interleukin 1 beta | *il1b* | 1.35 | 0.14 | -1.22 | 0.16 |
| Interleukin 6 | *il6* | 1.27 | 0.30 | -1.11 | 0.02 |
| Interleukin 10 | *il10* | -1.15 | 0.43 | 1.23 | 0.08 |
| Interleukin 10 receptor. alpha | *il10ra* | 1.23 | 0.10 | 1.13 | 0.19 |
| Interleukin 10 receptor. beta | *il10rb* | 1.75 | 0.004 | -1.80 | 0.004 |
| Interleukin 22 | *il22* | -1.01 | 0.18 | 1.06 | 0.57 |
| Neutrophilcytosolic factor 2 | *ncf2* | 1.44 | 0.013 | -1.54 | 0.01 |
| Nitric oxide synthase 2, inducible | *nos2* | -1.09 | 0.4 | 1.15 | 0.55 |
| Prostaglandin-endoperoxidesynthase 1 | *ptgs1* | 2.10 | 0.002 | -1.60 | 0.006 |
| Prostaglandin-endoperoxide synthase 2 | *ptgs2* | 1.08 | 0.20 | 1.04 | 0.17 |
| Tumor necrosis factor | *tnf* | -1.21 | 0.08 | 1.12 | 0.2 |
| TNF receptor superfamily, member 10b | *tnfrsf10b* | 1.05 | 0.11 | 1.25 | 0.22 |
| TNF receptor superfamily, member 1a | *tnfrsf1a* | 1.29 | 0.10 | -1.41 | 0.02 |
| Vascular endothelial growth factor A | *vegfa* | -1.41 | 0.14 | -1.08 | 0.12 |
| Vascular endothelial growth factor B | *vegfb* | 1.12 | 0.11 | -1.07 | 0.13 |
| Vascular endothelial growth factor C | *vegfc* | -1.31 | 0.12 | 1.19 | 0.20 |

**Supplementary table 3.** Liver expression of genes encoding proteins of hepatocyte cytoskeleton.

| **Protein** | ***Gene*** | **Hz vs WT**  **(Fold change)** | **P Value** | **Hz+IGF-1 vs Hz**  **(Fold change)** | **P Value** |
| --- | --- | --- | --- | --- | --- |
| Desmin | *des* | -1.11 | 0.21 | 1.15 | 0.11 |
| Glial fibrillary acidic protein | *gfap* | -1.29 | 0.02 | 1.06 | 0.22 |
| Lamin A | *lmna* | -1.15 | 0.13 | 1.06 | 0.24 |
| Nestin | *nes* | -1.02 | 0.18 | 1.28 | 0.07 |
| Pleckstrin 2 | *plek2* | 1.01 | 0.17 | 1.08 | 0.21 |
| Keratin 7 | *krt7* | 1.05 | 0.22 | 1.07 | 0.39 |
| Caveolin 2 | *cav2* | -1.29 | 0.09 | 1.22 | 0.09 |
| Vinculin | *vcl* | -1.49 | 0.003 | 1.31 | 0.06 |
| Filamin, alpha | *flna* | 1.45 | 0.03 | -1.33 | 0.05 |
| Filamin, beta | *flnb* | -1.61 | 0.001 | 1.10 | 0.25 |
| Transgelin | *tagln* | -1.28 | 0.02 | 1.14 | 0.18 |
| Ras homolog gene family, member A | *rhoa* | 1.41 | 0.03 | -1.61 | 0.004 |
| RAS-related C3 botulinum substrate 1 | *rac1* | 1.30 | 0.02 | -1.21 | 0.06 |
| Spectrin alpha 1 | *spna1* | -1.13 | 0.13 | 1.21 | 0.19 |
| Spectrin alpha 2 | *spna2* | -1.98 | 0.007 | 1.42 | 0.0001 |
| Spectrin beta 1 | *spnb1* | 1.26 | 0.025 | -1.46 | 0.01 |
| Talin 1 | *tln1* | 1.16 | 0.20 | 1.37 | 0.02 |
| Talin 2 | *tln2* | -1.06 | 0.18 | 1.32 | 0.06 |
| Cortactin | *cttn* | 1.38 | 0.02 | 1.05 | 0.39 |
| Microtubule-associated protein 1B | *mtap1b* | 1.05 | 0.19 | 1.10 | 0.33 |
| Microtubule-associated protein, RP/EB family, 1 | *mapre1* | 1.10 | 0.30 | 1.22 | 0.19 |
| Microtubule-associated protein, RP/EB family, 2 | *mapre2* | 1.33 | 0.02 | -1.66 | 0.001 |
| MAP/microtubule affinity-regulating kinase 2 | *mark2* | -1.52 | 0.01 | 1.34 | 0.05 |
| Serine/threonine kinase 11 | *stk11* | -1.38 | 0.019 | 1.36 | 0.05 |
| Myosin IA | *myo1a* | -1.04 | 0.20 | 1.44 | 0.006 |
| Myosin IB | *myo1b* | 1.43 | 0.05 | -1.37 | 0.03 |
| Myosin IC | *myo1c* | 1.09 | 0.25 | -1.00 | 0.30 |
| Myosin ID | *myo1d* | -1.33 | 0.05 | 1.08 | 0.23 |
| Myosin IE | *myo1e* | 1.00 | 0.35 | -1.09 | 0.22 |
| Myosin IF | *myo1f* | 1.03 | 0.24 | -1.41 | 0.04 |
| Myosin IG | *myo1g* | 1.18 | 0.31 | -1.14 | 0.33 |
| Myosin IIIA | *myo3a* | -1.20 | 0.06 | 1.18 | 0.11 |
| Myosin IIIB | *myo3b* | -1.03 | 0.26 | -1.07 | 0.30 |
| Myosin VB | *myo5b* | 1.58 | 0.003 | -1.27 | 0.06 |
| Myosin VC | *myo5c* | -1.15 | 0.13 | 1.15 | 0.09 |
| Myosin VI | *myo6* | 1.14 | 0.13 | -1.25 | 0.10 |
| Myosin VIIA | *myo7a* | 1.13 | 0.12 | -1.50 | 0.06 |
| Myosin VIIB | *myo7b* | 1.21 | 0.20 | -1.20 | 0.08 |
| Myosin ixa | *myo9a* | -1.31 | 0.03 | 1.40 | 0.05 |
| Myosin ixb | *myo9b* | 1.05 | 0.28 | 1.02 | 0.32 |
| Myosin X | *myo10* | 1.28 | 0.08 | -1.23 | 0.03 |
| Myosin XV | *myo15* | -1.18 | 0.14 | 1.08 | 0.09 |
| Myosin XVB | *myo15b* | 1.11 | 0.32 | -1.16 | 0.10 |
| Myosin XVI | *myo16* | 1.03 | 0.15 | 1.09 | 0.39 |
| Myosin XVIIIA | *myo18a* | -1.10 | 0.37 | 1.08 | 0.09 |
| Myosin xviiib | *myo18b* | 1.22 | 0.03 | -1.05 | 0.06 |
| Actin, alpha, cardiac muscle 1 | *actc1* | 1.02 | 0.40 | -1.09 | 0.09 |
| Actin, alpha 2, smooth muscle, aorta | *acta2(a-sma)* | 1.08 | 0.39 | 1.04 | 0.13 |
| Actin, beta | *actb* | 1.65 | 0.0006 | -1.58 | 0.0001 |
| Actinin alpha 1 | *actn1* | 1.27 | 0.37 | -1.09 | 0.05 |
| Actinin alpha 2 | *actn2* | -1.18 | 0.012 | 1.14 | 0.40 |
| Actinin alpha 3 | *actn3* | -1.09 | 0.03 | 1.03 | 0.08 |
| Actinin alpha 4 | *actn4* | -1.20 | 0.02 | -1.03 | 0.75 |
| Tubulin, alpha 1A | *tuba1a* | -1.20 | 0.19 | 1.02 | 0.67 |
| Tubulin, alpha 1B | *tuba1b* | 1.02 | 0.13 | -1.24 | 0.09 |
| Tubulin, alpha 1C | *tuba1c* | -1.28 | 0.32 | 1.15 | 0.02 |
| Tubulin, alpha 3A | *tuba3a* | -1.08 | 0.10 | 1.06 | 0.17 |
| Tubulin, alpha 4A | *tuba4a* | -1.56 | 0.007 | 1.30 | 0.009 |
| Tubulin, alpha 8 | *tuba8* | -1.23 | 0.19 | 1.48 | 0.002 |
| Tubulin, beta 1 | *tubb1* | -1.21 | 0.32 | 1.34 | 0.09 |
| Tubulin, beta 2A | *tubb2a* | -2.49 | 0.001 | 1.56 | 0.002 |
| Tubulin, beta 2B | *tubb2b* | -1.04 | 0.10 | 1.07 | 0.14 |
| Tubulin, beta 2C | *tubb2c* | -1.36 | 0.04 | 1.24 | 0.06 |
| Tubulin, beta 3 | *tubb3* | -1.01 | 0.94 | 1.36 | 0.02 |
| Tubulin, beta 4 | *tubb4* | -1.25 | 0.03 | 1.09 | 0.01 |
| Tubulin, beta 5 | *tubb5* | -1.10 | 0.10 | 1.02 | 0.13 |
| Tubulin, beta 6 | *tubb6* | -1.09 | 0.45 | -1.20 | 0.04 |
| Tubulin, delta 1 | *tubd1* | 1.02 | 0.13 | -1.05 | 0.09 |
| Epsilon-tubulin 1 | *tube1* | 1.01 | 0.03 | 1.05 | 0.08 |
| Tubulin, gamma 1 | *tubg1* | 1.23 | 0.19 | 1.26 | 0.18 |
| Vimentin | *vim* | 1.25 | 0.43 | -1.76 | 0.0019 |
| Keratin 8 | *krt8* | -1.07 | 0.09 | -1.10 | 0.10 |

**Supplementary table 4.** Liver microarray expression of genes related to tight junctions, adherent junctions, desmosomes and gap junctions.

| **Protein** | **Gene** | **Hz vs WT**  **(Fold change)** | **P Value** | **Hz+IGF-1 vs Hz**  **(Fold change)** | **P Value** |
| --- | --- | --- | --- | --- | --- |
| Claudin 1 | *cldn1* | -1.04 | 0.21 | -1.23 | 0.18 |
| Claudin 10 | *cldn10* | 1.06 | 0.23 | -1.03 | 0.13 |
| Claudin 11 | *cldn11* | 1.02 | 0.18 | 1.00 | 0.87 |
| Claudin 12 | *cldn12* | -1.13 | 0.12 | 1.29 | 0.09 |
| Claudin 14 | *cldn14* | 2.37 | 0.0006 | -1.78 | 0.0003 |
| Claudin 15 | *cldn15* | -1.10 | 0.019 | 1.14 | 0.19 |
| Claudin 16 | *cldn16* | -1.12 | 0.32 | 1.11 | 0.24 |
| Claudin 18 | *cldn18* | 1.12 | 0.35 | -1.01 | 0.75 |
| Claudin 19 | *cldn19* | -1.26 | 0.05 | 1.05 | 0.38 |
| Claudin 2 | *cldn2* | -1.17 | 0.03 | -1.06 | 0.38 |
| Claudin 3 | *cldn3* | -1.22 | 0.023 | 1.11 | 0.23 |
| Claudin 4 | *cldn4* | -1.23 | 0.06 | 1.13 | 0.19 |
| Claudin 5 | *cldn5* | 1.20 | 0.066 | -1.02 | 0.48 |
| Claudin 6 | *cldn6* | -1.07 | 0.18 | 1.14 | 0.32 |
| Claudin 7 | *cldn7* | 1.48 | 0.003 | -1.15 | 0.12 |
| Claudin 8 | *cldn8* | -1.15 | 0.13 | 1.22 | 0.09 |
| Claudin 9 | *cldn9* | -1.14 | 0.17 | 1.00 | 0.82 |
| Occludin | *ocln* | -1.07 | 0.23 | 1.01 | 0.75 |
| Endothelial cell-specific adhesion molecule | *esam* | 1.08 | 0.26 | -1.23 | 0.03 |
| Intercellular adhesion molecule 1 | *icam1* | 1.29 | 0.09 | -1.33 | 0.02 |
| Intercellular adhesion molecule 2 | *icam2* | 1.01 | 0.41 | -1.12 | 0.28 |
| Platelet/endothelial cell adhesion molecule 1 | *pecam1* | 1.15 | 0.14 | -1.07 | 0.35 |
| F11 receptor | *F11r* | -1.12 | 0.23 | -1.12 | 0.20 |
| Immunoglobulin superfamily, member 5 | *Igsf5* | 1.32 | 0.02 | -1.22 | 0.09 |
| Junction adhesion molecule 2 | *Jam2* | 1.23 | 0.03 | -1.60 | 0.006 |
| Junction adhesion molecule 3 | *Jam3* | 1.05 | 0.19 | 1.04 | 0.28 |
| Cadherin 1 | *cdh1* | -1.04 | 0.46 | *2.04* | 0.01 |
| Cadherin 2 | *cdh2* | -1.01 | 0.47 | 1.01 | 0.65 |
| Cadherin 3 | *cdh3* | 1.02 | 0.45 | -1.29 | 0.32 |
| Cadherin 4 | *cdh4* | -1.16 | 0.4 | 1.09 | 0.03 |
| *Cadherin 5* | *cdh5* | *1.70* | 0.0006 | *-1.80* | 0.0001 |
| Desmocollin 1 | *dsc1* | 1.01 | 0.67 | 1.09 | 0.027 |
| *Desmocollin 2* | *dsc2* | *1.46* | 0.003 | *-1.46* | 0.001 |
| Desmocollin 3 | *dsc3* | 1.17 | 0.08 | 1.01 | 0.68 |
| Desmoglein 1 alpha | *dsg1a* | -1.16 | 0.03 | -1.00 | 0.89 |
| Desmoglein 1 beta | *dsg1b* | 1.01 | 0.63 | -1.03 | 0.55 |
| Desmoglein 2 | *dsg2* | -1.27 | 0.35 | -1.28 | 0.41 |
| Catenin (cadherin associated protein), alpha 1 | *ctnna1* | -1.12 | 0.21 | -1.16 | 0.12 |
| Catenin (cadherin associated protein), alpha 2 | *ctnna2* | -1.02 | 0.43 | -1.02 | 0.35 |
| Catenin (cadherin associated protein), alpha 3 | *ctnna3* | -1.23 | 0.13 | 1.14 | 0.18 |
| Catenin (cadherin associated protein), beta 1 | *ctnnb1* | 1.25 | 0.09 | -1.33 | 0.08 |
| Catenin (cadherin associated protein), delta 1 | *ctnnd1* | -1.20 | 0.15 | 1.07 | 0.45 |
| *Desmoplakin* | *dsp* | *1.53* | 0.01 | 1.04 | 0.43 |
| Junction plakoglobin | *jup* | 1.03 | 0.33 | -1.05 | 0.62 |
| Plakophilin 1 | *pkp1* | -1.24 | 0.13 | 1.21 | 0.08 |
| Plakophilin 2 | *pkp2* | -1.06 | 0.24 | 1.15 | 0.10 |
| Plakophilin 3 | *pkp3* | 1.16 | 0.11 | 1.08 | 0.21 |
| Plakophilin 4 | *pkp4* | -1.31 | 0.06 | 1.10 | 0.21 |
| Pinin | *pnn* | -1.09 | 0.22 | -1.15 | 0.16 |
| Periplakin | *ppl* | 1.20 | 0.18 | -1.20 | 0.08 |
| Gap junction protein, alpha 1 | *gja1* | -1.23 | 0.09 | 1.17 | 0.19 |
| Gap junction protein, alpha 3 | *gja3* | 1.04 | 0.42 | 1.15 | 0.13 |
| Gap junction protein, alpha 4 | *gja4* | 1.11 | 0.32 | -1.10 | 0.30 |
| Gap junction protein, alpha 5 | *gja5* | 1.07 | 0.19 | -1.05 | 0.46 |
| Gap junction protein, alpha 8 | *gja8* | -1.01 | 0.38 | 1.20 | 0.09 |
| Gap junction protein, beta 1 | *gjb1* | 1.11 | 0.45 | 1.15 | 0.46 |
| Gap junction protein, beta 2 | *gjb2* | 1.18 | 0.25 | -1.37 | 0.05 |
| Gap junction protein, beta 3 | *gjb3* | 1.07 | 0.35 | -1.02 | 0.41 |
| Gap junction protein, beta 4 | *gjb4* | 1.11 | 0.22 | -1.03 | 0.38 |
| Gap junction protein, beta 5 | *gjb5* | 1.02 | 0.47 | 1.14 | 0.19 |
| Gap junction protein, beta 6 | *gjb6* | -1.11 | 0.23 | 1.00 | 0.60 |
| Gap junction protein, gamma 2 | *gjc2* | -1.24 | 0.07 | 1.20 | 0.09 |
| Gap junction protein, gamma 3 | *gjc3* | -1.04 | 0.26 | -1.11 | 0.21 |
| Gap junction protein, delta 2 | *gjd2* | 1.19 | 0.15 | -1.00 | 0.29 |
| Gap junction protein, epsilon 1 | *gje1* | 1.09 | 0.29 | -1.04 | 0.32 |
| Pannexin 1 | *panx1* | -1.12 | 0.20 | -1.09 | 0.37 |
| Pannexin 2 | *panx2* | 1.19 | 0.37 | 1.12 | 0.24 |
| Pannexin 3 | *panx3* | -1.15 | 0.09 | 1.12 | 0.21 |
