# Supplementary material for: IGF-1 modulates gene expression of proteins involved in inflammation, cytoskeleton, and liver architecture
Source: J Physiol Biochem. 2017 Jan 26;73(2):245–58. doi: 10.1007/s13105-016-0545-x (PMC5399066; doi:10.1007/s13105-016-0545-x)
Supplement: Supplementary file 2 — (DOCX 101 kb) [file 13105_2016_545_MOESM2_ESM.docx]

**Supplementary table 2.** Liver expression of genes encoding proteins involved in inflammatory and acute-phase response proteins.

| **Protein** | ***Gene*** | **Hz vs WT**  **(Fold change)** | **P Value** | **Hz+IGF-1 vs Hz**  **(Fold change)** | **P Value** |
| --- | --- | --- | --- | --- | --- |
| Apoptotic peptidase activating factor 1 | *apaf1* | 1.05 | 0.05 | -1.05 | 0.05 |
| Allograft inflammatory factor 1 | *aif1* | 1.95 | 0.0001 | -2.82 | 0.00006 |
| Colony stimulating factor 1 (macrophage) | *csf1* | 1.09 | 0.46 | 1.29 | 0.16 |
| Colony stimulating factor 1 receptor | *csf1r* | 2.49 | 0.0004 | -2.41 | 0.0006 |
| Colony stimulating factor 3 receptor (granulocyte) | *csf3r* | 1.04 | 0.37 | 1.08 | 0.41 |
| Colony stimulating factor 3 (granulocyte) | *csf3* | 1.18 | 0.12 | 1.02 | 0.52 |
| Colony stimulating factor 2 | *csf2* | -1.03 | 0.11 | 1.16 | 0.19 |
| Colony stimulating factor 2 receptor alpha | *csf2ra* | 1.35 | 0.018 | -1.30 | 0.019 |
| Colony stimulating factor 2 receptor beta 2 | *csf2rb2* | 2.38 | 0.0001 | -2.39 | 0.0006 |
| Interferon gamma receptor 1 | *ifngr1* | 1.62 | 0.0003 | -1.34 | 0.06 |
| Chemokine (C-C motif) ligand 11 | *ccl11* | 1.12 | 0.07 | -1.01 | 0.47 |
| Chemokine (C-C motif) receptor 6 | *ccr6* | -1.10 | 0.18 | 1.00 | 0.5 |
| Chemokine (C-C motif) ligand 6 | *ccl6* | 4.34 | 0.0002 | -4.02 | 0.0001 |
| Chemokine (C-C motif) receptor 5 | *ccr5* | 4.38 | 0.0001 | -4.87 | 0.0001 |
| Chemokine (C-C motif) ligand 12 | *ccl12* | 1.10 | 0.13 | -1.02 | 0.48 |
| Chemokine (C-C motif) ligand 5 | *ccl5* | 1.40 | 0.017 | -1.06 | 0.33 |
| Chemokine (C-C motif) ligand 17 | *ccl17* | 1.26 | 0.17 | -1.20 | 0.19 |
| Chemokine (C-C motif) ligand 3 | *ccl3* | -1.28 | 0.133 | -1.12 | 0.37 |
| Chemokine (C-C motif) ligand 4 | *ccl4* | 1.06 | 0.19 | 1.07 | 0.35 |
| Chemokine (C-X-C motif) ligand 1 | *cxcl1* | 1.66 | 0.0006 | -2.13 | 0.002 |
| Chemokine (C-X-C motif) ligand 14 | *cxcl14* | 4.06 | 0.00001 | -1.11 | 0.21 |
| Chemokine (C-X-C motif) ligand 16 | *cxcl16* | 1.43 | 0.03 | -1.58 | 0.03 |
| Interferon alpha 2 | *ifna2* | 1.06 | 0.11 | -1.05 | 0.28 |
| Interleukin 12a | *il12a* | 1.16 | 0.35 | 1.02 | 0.33 |
| Interleukin 12b | *il12b* | 1.23 | 0.019 | 1.09 | 0.20 |
| Interleukin 13 | *il13* | 1.06 | 0.38 | 1.11 | 0.15 |
| Interleukin 13 receptor alpha 1 | *il13ra1* | 1.43 | 0.01 | -1.61 | 0.005 |
| Leukemia inhibitory factor | *lif* | 1.03 | 0.42 | 1.06 | 0.42 |
| Oncostatin M receptor | *osmr* | 1.54 | 0.005 | -1.56 | 0.03 |
| Oncostatin M | *osm* | 1.15 | 0.42 | 1.00 | 0.28 |
| Secreted phosphoprotein 1 | *spp1* | 2.44 | 0.001 | -1.80 | 0.005 |
| Lysozyme 1 | *lyz1* | 3.26 | 0.0002 | -2.79 | 0.0017 |
| Lysozyme 2 | *lyz2* | 2.99 | 0.0019 | -2.31 | 0.004 |
| Solute carrier family 11 | *slc11a1* | 1.45 | 0.018 | -1.50 | 0.025 |
| Toll-like receptor 1 | *tlr1* | 1.52 | 0.01 | -1.40 | 0.28 |
| Lymphocyte antigen 96 | *ly96* | 1.71 | 0.003 | -1.38 | 0.04 |
| Complement component 3 | *c3* | 1.89 | 0.002 | -1.57 | 0.002 |
| Complement component 1, q subcomponent, α p | *c1qa* | 5.40 | 0.0002 | -3.43 | 0.0006 |
| Complement component 1, q subcomponent, ß p | *c1qb* | 6.61 | 0.0001 | -2.43 | 0.001 |
| Complement component 1, q subcomponent, C | *c1qc* | 3.32 | 0.0006 | -2.69 | 0.0006 |
| Macrophage activation 2 like | *mpa2l* | 3.11 | 0.0006 | -2.62 | 0.0001 |
| Orosomucoid 1 | *orm1* | 3.51 | 0.0017 | -2.44 | 0.0002 |
| Orosomucoid 2 | *orm2* | 10.52 | 0.0001 | -6.49 | 0.0001 |
| S100 calcium binding protein A8 (calgranulin A) | *s100a8* | 2.89 | 0.002 | 1.16 | 0.4 |
| S100 calcium binding protein A9 (calgranulin B) | *s100a9* | 2.00 | 0.002 | 1.20 | 0.14 |
| Serum amyloid A 1 | *saa1* | 3.74 | 0.0004 | -1.70 | 0.006 |
| Serum amyloid A 2 | *saa2* | 4.52 | 0.0004 | -2.62 | 0.0002 |
| Serum amyloid A 3 | *saa3* | 1.72 | 0.012 | -1.31 | 0.02 |
| Serum amyloid A 4 | *saa4* | 1.29 | 0.15 | -1.24 | 0.12 |
| Histocompatibility 2, class II antigen A, alpha | *h2-aa* | 6.56 | 0.0001 | -4.22 | 0.001 |
| Histocompatibility 2, class II antigen A, beta 1 | *h2-ab1* | 2.40 | 0.001 | -4.22 | 0.002 |
| Histocompatibility 2, class II antigen E beta | *h2-eb1* | 3.17 | 0.0002 | -2.97 | 0.001 |
| Arginase, liver | *arg1* | 1.33 | 0.13 | -1.37 | 0.08 |
| Eosinophilperoxidase | *epx* | -1.01 | 0.47 | 1.35 | 0.10 |
| Lactoperoxidase | *lpo* | -1.17 | 0.33 | 1.16 | 0.19 |
| Monoamine oxidase A | *maoa* | 1.06 | 0.37 | -1.20 | 0.21 |
| Monoamine oxidase B | *maob* | 1.11 | 0.33 | -1.24 | 0.09 |
| Peroxidasin homolog (Drosophila) | *pxdn* | 1.03 | 0.11 | -1.05 | 0.12 |
| Xanthine dehydrogenase | *xdh* | 1.42 | 0.03 | -1.34 | 0.14 |
| Interferon gamma | *ifng* | -1.13 | 0.34 | 1.12 | 0.38 |
| Interleukin 1 alpha | *il1a* | -1.01 | 0.54 | 1.04 | 0.57 |
| Interleukin 1 beta | *il1b* | 1.35 | 0.14 | -1.22 | 0.16 |
| Interleukin 6 | *il6* | 1.27 | 0.30 | -1.11 | 0.02 |
| Interleukin 10 | *il10* | -1.15 | 0.43 | 1.23 | 0.08 |
| Interleukin 10 receptor. alpha | *il10ra* | 1.23 | 0.10 | 1.13 | 0.19 |
| Interleukin 10 receptor. beta | *il10rb* | 1.75 | 0.004 | -1.80 | 0.004 |
| Interleukin 22 | *il22* | -1.01 | 0.18 | 1.06 | 0.57 |
| Neutrophilcytosolic factor 2 | *ncf2* | 1.44 | 0.013 | -1.54 | 0.01 |
| Nitric oxide synthase 2, inducible | *nos2* | -1.09 | 0.4 | 1.15 | 0.55 |
| Prostaglandin-endoperoxidesynthase 1 | *ptgs1* | 2.10 | 0.002 | -1.60 | 0.006 |
| Prostaglandin-endoperoxide synthase 2 | *ptgs2* | 1.08 | 0.20 | 1.04 | 0.17 |
| Tumor necrosis factor | *tnf* | -1.21 | 0.08 | 1.12 | 0.2 |
| TNF receptor superfamily, member 10b | *tnfrsf10b* | 1.05 | 0.11 | 1.25 | 0.22 |
| TNF receptor superfamily, member 1a | *tnfrsf1a* | 1.29 | 0.10 | -1.41 | 0.02 |
| Vascular endothelial growth factor A | *vegfa* | -1.41 | 0.14 | -1.08 | 0.12 |
| Vascular endothelial growth factor B | *vegfb* | 1.12 | 0.11 | -1.07 | 0.13 |
| Vascular endothelial growth factor C | *vegfc* | -1.31 | 0.12 | 1.19 | 0.20 |
