# Supplementary material for: IGF-1 modulates gene expression of proteins involved in inflammation, cytoskeleton, and liver architecture
Source: J Physiol Biochem. 2017 Jan 26;73(2):245–58. doi: 10.1007/s13105-016-0545-x (PMC5399066; doi:10.1007/s13105-016-0545-x)
Supplement: Supplementary file 3 — (DOCX 94 kb) [file 13105_2016_545_MOESM3_ESM.docx]

**Supplementary table 3.** Liver expression of genes encoding proteins of hepatocyte cytoskeleton.

| **Protein** | ***Gene*** | **Hz vs WT**  **(Fold change)** | **P Value** | **Hz+IGF-1 vs Hz**  **(Fold change)** | **P Value** |
| --- | --- | --- | --- | --- | --- |
| Desmin | *des* | -1.11 | 0.21 | 1.15 | 0.11 |
| Glial fibrillary acidic protein | *gfap* | -1.29 | 0.02 | 1.06 | 0.22 |
| Lamin A | *lmna* | -1.15 | 0.13 | 1.06 | 0.24 |
| Nestin | *nes* | -1.02 | 0.18 | 1.28 | 0.07 |
| Pleckstrin 2 | *plek2* | 1.01 | 0.17 | 1.08 | 0.21 |
| Keratin 7 | *krt7* | 1.05 | 0.22 | 1.07 | 0.39 |
| Caveolin 2 | *cav2* | -1.29 | 0.09 | 1.22 | 0.09 |
| Vinculin | *vcl* | -1.49 | 0.003 | 1.31 | 0.06 |
| Filamin, alpha | *flna* | 1.45 | 0.03 | -1.33 | 0.05 |
| Filamin, beta | *flnb* | -1.61 | 0.001 | 1.10 | 0.25 |
| Transgelin | *tagln* | -1.28 | 0.02 | 1.14 | 0.18 |
| Ras homolog gene family, member A | *rhoa* | 1.41 | 0.03 | -1.61 | 0.004 |
| RAS-related C3 botulinum substrate 1 | *rac1* | 1.30 | 0.02 | -1.21 | 0.06 |
| Spectrin alpha 1 | *spna1* | -1.13 | 0.13 | 1.21 | 0.19 |
| Spectrin alpha 2 | *spna2* | -1.98 | 0.007 | 1.42 | 0.0001 |
| Spectrin beta 1 | *spnb1* | 1.26 | 0.025 | -1.46 | 0.01 |
| Talin 1 | *tln1* | 1.16 | 0.20 | 1.37 | 0.02 |
| Talin 2 | *tln2* | -1.06 | 0.18 | 1.32 | 0.06 |
| Cortactin | *cttn* | 1.38 | 0.02 | 1.05 | 0.39 |
| Microtubule-associated protein 1B | *mtap1b* | 1.05 | 0.19 | 1.10 | 0.33 |
| Microtubule-associated protein, RP/EB family, 1 | *mapre1* | 1.10 | 0.30 | 1.22 | 0.19 |
| Microtubule-associated protein, RP/EB family, 2 | *mapre2* | 1.33 | 0.02 | -1.66 | 0.001 |
| MAP/microtubule affinity-regulating kinase 2 | *mark2* | -1.52 | 0.01 | 1.34 | 0.05 |
| Serine/threonine kinase 11 | *stk11* | -1.38 | 0.019 | 1.36 | 0.05 |
| Myosin IA | *myo1a* | -1.04 | 0.20 | 1.44 | 0.006 |
| Myosin IB | *myo1b* | 1.43 | 0.05 | -1.37 | 0.03 |
| Myosin IC | *myo1c* | 1.09 | 0.25 | -1.00 | 0.30 |
| Myosin ID | *myo1d* | -1.33 | 0.05 | 1.08 | 0.23 |
| Myosin IE | *myo1e* | 1.00 | 0.35 | -1.09 | 0.22 |
| Myosin IF | *myo1f* | 1.03 | 0.24 | -1.41 | 0.04 |
| Myosin IG | *myo1g* | 1.18 | 0.31 | -1.14 | 0.33 |
| Myosin IIIA | *myo3a* | -1.20 | 0.06 | 1.18 | 0.11 |
| Myosin IIIB | *myo3b* | -1.03 | 0.26 | -1.07 | 0.30 |
| Myosin VB | *myo5b* | 1.58 | 0.003 | -1.27 | 0.06 |
| Myosin VC | *myo5c* | -1.15 | 0.13 | 1.15 | 0.09 |
| Myosin VI | *myo6* | 1.14 | 0.13 | -1.25 | 0.10 |
| Myosin VIIA | *myo7a* | 1.13 | 0.12 | -1.50 | 0.06 |
| Myosin VIIB | *myo7b* | 1.21 | 0.20 | -1.20 | 0.08 |
| Myosin ixa | *myo9a* | -1.31 | 0.03 | 1.40 | 0.05 |
| Myosin ixb | *myo9b* | 1.05 | 0.28 | 1.02 | 0.32 |
| Myosin X | *myo10* | 1.28 | 0.08 | -1.23 | 0.03 |
| Myosin XV | *myo15* | -1.18 | 0.14 | 1.08 | 0.09 |
| Myosin XVB | *myo15b* | 1.11 | 0.32 | -1.16 | 0.10 |
| Myosin XVI | *myo16* | 1.03 | 0.15 | 1.09 | 0.39 |
| Myosin XVIIIA | *myo18a* | -1.10 | 0.37 | 1.08 | 0.09 |
| Myosin xviiib | *myo18b* | 1.22 | 0.03 | -1.05 | 0.06 |
| Actin, alpha, cardiac muscle 1 | *actc1* | 1.02 | 0.40 | -1.09 | 0.09 |
| Actin, alpha 2, smooth muscle, aorta | *acta2(a-sma)* | 1.08 | 0.39 | 1.04 | 0.13 |
| Actin, beta | *actb* | 1.65 | 0.0006 | -1.58 | 0.0001 |
| Actinin alpha 1 | *actn1* | 1.27 | 0.37 | -1.09 | 0.05 |
| Actinin alpha 2 | *actn2* | -1.18 | 0.012 | 1.14 | 0.40 |
| Actinin alpha 3 | *actn3* | -1.09 | 0.03 | 1.03 | 0.08 |
| Actinin alpha 4 | *actn4* | -1.20 | 0.02 | -1.03 | 0.75 |
| Tubulin, alpha 1A | *tuba1a* | -1.20 | 0.19 | 1.02 | 0.67 |
| Tubulin, alpha 1B | *tuba1b* | 1.02 | 0.13 | -1.24 | 0.09 |
| Tubulin, alpha 1C | *tuba1c* | -1.28 | 0.32 | 1.15 | 0.02 |
| Tubulin, alpha 3A | *tuba3a* | -1.08 | 0.10 | 1.06 | 0.17 |
| Tubulin, alpha 4A | *tuba4a* | -1.56 | 0.007 | 1.30 | 0.009 |
| Tubulin, alpha 8 | *tuba8* | -1.23 | 0.19 | 1.48 | 0.002 |
| Tubulin, beta 1 | *tubb1* | -1.21 | 0.32 | 1.34 | 0.09 |
| Tubulin, beta 2A | *tubb2a* | -2.49 | 0.001 | 1.56 | 0.002 |
| Tubulin, beta 2B | *tubb2b* | -1.04 | 0.10 | 1.07 | 0.14 |
| Tubulin, beta 2C | *tubb2c* | -1.36 | 0.04 | 1.24 | 0.06 |
| Tubulin, beta 3 | *tubb3* | -1.01 | 0.94 | 1.36 | 0.02 |
| Tubulin, beta 4 | *tubb4* | -1.25 | 0.03 | 1.09 | 0.01 |
| Tubulin, beta 5 | *tubb5* | -1.10 | 0.10 | 1.02 | 0.13 |
| Tubulin, beta 6 | *tubb6* | -1.09 | 0.45 | -1.20 | 0.04 |
| Tubulin, delta 1 | *tubd1* | 1.02 | 0.13 | -1.05 | 0.09 |
| Epsilon-tubulin 1 | *tube1* | 1.01 | 0.03 | 1.05 | 0.08 |
| Tubulin, gamma 1 | *tubg1* | 1.23 | 0.19 | 1.26 | 0.18 |
| Vimentin | *vim* | 1.25 | 0.43 | -1.76 | 0.0019 |
| Keratin 8 | *krt8* | -1.07 | 0.09 | -1.10 | 0.10 |
