# Supplementary material for: IGF-1 modulates gene expression of proteins involved in inflammation, cytoskeleton, and liver architecture
Source: J Physiol Biochem. 2017 Jan 26;73(2):245–58. doi: 10.1007/s13105-016-0545-x (PMC5399066; doi:10.1007/s13105-016-0545-x)
Supplement: Supplementary file 4 — (DOCX 105 kb) [file 13105_2016_545_MOESM4_ESM.docx]

**Supplementary table 4.** Liver microarray expression of genes related to tight junctions, adherent junctions, desmosomes and gap junctions.

| **Protein** | **Gene** | **Hz vs WT**  **(Fold change)** | **P Value** | **Hz+IGF-1 vs Hz**  **(Fold change)** | **P Value** |
| --- | --- | --- | --- | --- | --- |
| Claudin 1 | *cldn1* | -1.04 | 0.21 | -1.23 | 0.18 |
| Claudin 10 | *cldn10* | 1.06 | 0.23 | -1.03 | 0.13 |
| Claudin 11 | *cldn11* | 1.02 | 0.18 | 1.00 | 0.87 |
| Claudin 12 | *cldn12* | -1.13 | 0.12 | 1.29 | 0.09 |
| Claudin 14 | *cldn14* | 2.37 | 0.0006 | -1.78 | 0.0003 |
| Claudin 15 | *cldn15* | -1.10 | 0.019 | 1.14 | 0.19 |
| Claudin 16 | *cldn16* | -1.12 | 0.32 | 1.11 | 0.24 |
| Claudin 18 | *cldn18* | 1.12 | 0.35 | -1.01 | 0.75 |
| Claudin 19 | *cldn19* | -1.26 | 0.05 | 1.05 | 0.38 |
| Claudin 2 | *cldn2* | -1.17 | 0.03 | -1.06 | 0.38 |
| Claudin 3 | *cldn3* | -1.22 | 0.023 | 1.11 | 0.23 |
| Claudin 4 | *cldn4* | -1.23 | 0.06 | 1.13 | 0.19 |
| Claudin 5 | *cldn5* | 1.20 | 0.066 | -1.02 | 0.48 |
| Claudin 6 | *cldn6* | -1.07 | 0.18 | 1.14 | 0.32 |
| Claudin 7 | *cldn7* | 1.48 | 0.003 | -1.15 | 0.12 |
| Claudin 8 | *cldn8* | -1.15 | 0.13 | 1.22 | 0.09 |
| Claudin 9 | *cldn9* | -1.14 | 0.17 | 1.00 | 0.82 |
| Occludin | *ocln* | -1.07 | 0.23 | 1.01 | 0.75 |
| Endothelial cell-specific adhesion molecule | *esam* | 1.08 | 0.26 | -1.23 | 0.03 |
| Intercellular adhesion molecule 1 | *icam1* | 1.29 | 0.09 | -1.33 | 0.02 |
| Intercellular adhesion molecule 2 | *icam2* | 1.01 | 0.41 | -1.12 | 0.28 |
| Platelet/endothelial cell adhesion molecule 1 | *pecam1* | 1.15 | 0.14 | -1.07 | 0.35 |
| F11 receptor | *F11r* | -1.12 | 0.23 | -1.12 | 0.20 |
| Immunoglobulin superfamily, member 5 | *Igsf5* | 1.32 | 0.02 | -1.22 | 0.09 |
| Junction adhesion molecule 2 | *Jam2* | 1.23 | 0.03 | -1.60 | 0.006 |
| Junction adhesion molecule 3 | *Jam3* | 1.05 | 0.19 | 1.04 | 0.28 |
| Cadherin 1 | *cdh1* | -1.04 | 0.46 | *2.04* | 0.01 |
| Cadherin 2 | *cdh2* | -1.01 | 0.47 | 1.01 | 0.65 |
| Cadherin 3 | *cdh3* | 1.02 | 0.45 | -1.29 | 0.32 |
| Cadherin 4 | *cdh4* | -1.16 | 0.4 | 1.09 | 0.03 |
| *Cadherin 5* | *cdh5* | *1.70* | 0.0006 | *-1.80* | 0.0001 |
| Desmocollin 1 | *dsc1* | 1.01 | 0.67 | 1.09 | 0.027 |
| *Desmocollin 2* | *dsc2* | *1.46* | 0.003 | *-1.46* | 0.001 |
| Desmocollin 3 | *dsc3* | 1.17 | 0.08 | 1.01 | 0.68 |
| Desmoglein 1 alpha | *dsg1a* | -1.16 | 0.03 | -1.00 | 0.89 |
| Desmoglein 1 beta | *dsg1b* | 1.01 | 0.63 | -1.03 | 0.55 |
| Desmoglein 2 | *dsg2* | -1.27 | 0.35 | -1.28 | 0.41 |
| Catenin (cadherin associated protein), alpha 1 | *ctnna1* | -1.12 | 0.21 | -1.16 | 0.12 |
| Catenin (cadherin associated protein), alpha 2 | *ctnna2* | -1.02 | 0.43 | -1.02 | 0.35 |
| Catenin (cadherin associated protein), alpha 3 | *ctnna3* | -1.23 | 0.13 | 1.14 | 0.18 |
| Catenin (cadherin associated protein), beta 1 | *ctnnb1* | 1.25 | 0.09 | -1.33 | 0.08 |
| Catenin (cadherin associated protein), delta 1 | *ctnnd1* | -1.20 | 0.15 | 1.07 | 0.45 |
| *Desmoplakin* | *dsp* | *1.53* | 0.01 | 1.04 | 0.43 |
| Junction plakoglobin | *jup* | 1.03 | 0.33 | -1.05 | 0.62 |
| Plakophilin 1 | *pkp1* | -1.24 | 0.13 | 1.21 | 0.08 |
| Plakophilin 2 | *pkp2* | -1.06 | 0.24 | 1.15 | 0.10 |
| Plakophilin 3 | *pkp3* | 1.16 | 0.11 | 1.08 | 0.21 |
| Plakophilin 4 | *pkp4* | -1.31 | 0.06 | 1.10 | 0.21 |
| Pinin | *pnn* | -1.09 | 0.22 | -1.15 | 0.16 |
| Periplakin | *ppl* | 1.20 | 0.18 | -1.20 | 0.08 |
| Gap junction protein, alpha 1 | *gja1* | -1.23 | 0.09 | 1.17 | 0.19 |
| Gap junction protein, alpha 3 | *gja3* | 1.04 | 0.42 | 1.15 | 0.13 |
| Gap junction protein, alpha 4 | *gja4* | 1.11 | 0.32 | -1.10 | 0.30 |
| Gap junction protein, alpha 5 | *gja5* | 1.07 | 0.19 | -1.05 | 0.46 |
| Gap junction protein, alpha 8 | *gja8* | -1.01 | 0.38 | 1.20 | 0.09 |
| Gap junction protein, beta 1 | *gjb1* | 1.11 | 0.45 | 1.15 | 0.46 |
| Gap junction protein, beta 2 | *gjb2* | 1.18 | 0.25 | -1.37 | 0.05 |
| Gap junction protein, beta 3 | *gjb3* | 1.07 | 0.35 | -1.02 | 0.41 |
| Gap junction protein, beta 4 | *gjb4* | 1.11 | 0.22 | -1.03 | 0.38 |
| Gap junction protein, beta 5 | *gjb5* | 1.02 | 0.47 | 1.14 | 0.19 |
| Gap junction protein, beta 6 | *gjb6* | -1.11 | 0.23 | 1.00 | 0.60 |
| Gap junction protein, gamma 2 | *gjc2* | -1.24 | 0.07 | 1.20 | 0.09 |
| Gap junction protein, gamma 3 | *gjc3* | -1.04 | 0.26 | -1.11 | 0.21 |
| Gap junction protein, delta 2 | *gjd2* | 1.19 | 0.15 | -1.00 | 0.29 |
| Gap junction protein, epsilon 1 | *gje1* | 1.09 | 0.29 | -1.04 | 0.32 |
| Pannexin 1 | *panx1* | -1.12 | 0.20 | -1.09 | 0.37 |
| Pannexin 2 | *panx2* | 1.19 | 0.37 | 1.12 | 0.24 |
| Pannexin 3 | *panx3* | -1.15 | 0.09 | 1.12 | 0.21 |
